# Microglia-independent rAAV-induced inflammation causes persistent ocular immune dysregulation rescued by S1P receptor modulation

**DOI:** 10.1016/j.ymthe.2026.02.018

**Published:** 2026-03-13

**Authors:** Philip M. Langer, Alison J. Clare, Xudong Peng, Katherine L. Costello, Suci Cendanawati, Leslie L. Wilson, Amy Ward, Oliver H. Bell, Colin J. Chu, Ying Kai Chan, Andrew D. Dick, Kathryn L. Pepple, David A. Copland

**Affiliations:** 1 Academic Unit of Ophthalmology, Bristol Medical School, University of Bristol, Bristol BS8 1TD, UK;; 2 Department of Ophthalmology, School of Medicine, University of Washington, Seattle, WA 98104, USA;; 3 School of Cellular and Molecular Medicine, University of Bristol, Bristol BS8 1TD, UK;; 4 Institute of Ophthalmology, University College London, London EC1V 9EL, UK;; 5 Wyss Institute for Biologically Inspired Engineering, Harvard University, Boston, MA 02215, USA;; 6 National Institute of Health Research (NIHR) Moorfields Biomedical Research Centre, Moorfields Eye Hospital NHS Foundation Trust and University College London Institute of Ophthalmology, London EC1V 2PD, UK

## Abstract

Inflammation elicited by rAAV vectors continues to present a critical challenge for the long-term efficacy and safety of gene therapy in the eye. Preclinical models of gene therapy-associated uveitis (GTAU) show that despite the resolution of early acute inflammatory response, persistent subclinical inflammation remains. Here, we employ the GTAU model in *Cx3cr1*^CreER^:*R26-tdTomato*^+/−^ mice to reveal that intravitreal rAAV2 administration elicits sustained microglial dysregulation and retention of CD3^+^ T cells extending to 50 days post-injection. Deploying pharmacologic and genetic approaches, we define the absolute requirement for microglia and T cells to mediate rAAV2-induced inflammation. Targeted depletion confirmed that microglia-independent mechanisms initiate GTAU, while elimination of lymphocytes prevented both inflammation and microglial activation. Systematic evaluation of therapeutic strategies reveals identified inhibition of T cell recruitment via sphingosine-1-phosphate receptor modulation, but not B cell depletion, as an effective steroid-sparing strategy to prevent both acute and long-term subclinical inflammation. Collectively, our findings challenge the paradigm of microglia-driven ocular inflammation and support the utility of targeted T cell immunomodulation strategies to control GTAU and maintain long-term ocular homeostasis.

## INTRODUCTION

Recombinant adeno-associated virus (rAAV) vectors are the leading delivery platform for ocular gene therapies due to their broad tissue tropism, stable non-integrating transduction, and reduced immunogenicity.^1–3^ However, preclinical and clinical studies report dose-dependent rAAV-induced inflammation, termed gene therapy-associated uveitis (GTAU), which limits dose escalation and therapeutic efficacy.^4–7^ The incidence and extent of the GTAU response is dependent on the route of vector administration, highlighted by recent meta-analysis of clinical gene therapy trials showing that GTAU occurs in around 45% of patients receiving intravitreal (IVT) gene therapy, compared with 28% after subretinal gene therapy and 21% after suprachoroidal gene therapy (the latter with very limited data to date).^8^ In some patients, severe cases of GTAU are linked to permanent loss of visual acuity, and overall inflammation is impacting outcomes, with long-term durability achieved in only 43.6% of ocular gene therapy trials.^9–14^ Current clinical management of rAAV-induced inflammation primarily relies on prophylactic or reactive corticosteroid treatment,^15^ which produces mixed efficacy while causing significant adverse effects that can limit their use clinically, particularly in target pediatric and geriatric populations.^16–19^ Hence, there is a pressing need to improve our understanding of the cellular response to rAAV to inform new strategies to mitigate clinical and subclinical inflammation and enhance therapeutic efficacy.

Characterization of the acute immune response in preclinical models shows that administration of rAAV vectors in the eye evokes both innate and adaptive immune responses, as well as recruitment of peripheral CD45^+^ leukocytes to the ocular tissue.^5,20–23^ While the early peak of clinically evident inflammation following IVT rAAV injection can spontaneously resolve, *ex vivo* assessment of ocular tissues highlights persistent subclinical inflammation, with an elevated number of immune cells remaining within rAAV-exposed tissue. During both peak and later subclinical stages of GTAU, the predominant infiltrating cells are CD3^+^ lymphocytes, comprising CD4^+^ and CD8^+^ populations. Recent work demonstrates the importance of the posterior ocular lymphatic drainage system in the recruitment of rAAV-specific CD8^+^ T cells to the eye, but the functional role of these potentially cytotoxic cells in GTAU has not been determined.^24^

The initiation and amplification of innate responses in the CNS, of which the neural retina is a part, have focused on the interaction between the long-lived, yolk sac-derived microglia and infiltrating T cells.^25–28^ In the retina, microglia reside within two functionally distinct niches,^29^ expressing Toll-like receptors (TLRs) 2, 4, and 9,^30,31^ which detect capsid protein and unmethylated CpG motifs and possess phagocytic and antigen presentation capabilities to maintain homeostasis.^32–34^ Evidence from preclinical models demonstrates that rAAV induces activation of microglia, with changes to their morphology, tissue distribution, and transcriptome.^5,20,23,35^ Understanding the plasticity of microglial response will inform the extent to which homeostatic pathways are perturbed and whether or not the microglia initiate and/or amplify the acute and persistent stages of inflammation.

Extending from our recent work,^20^ we use a combination of flow cytometry and transcriptomics in *Cx3cr1*^*CreER*^*:R26-tdTomato*^+/−^ microglia reporter mice to provide an in-depth characterization of the early acute inflammation-responsive transition,^36^ as well as long-term phenotypic changes of microglia to IVT rAAV2 administration. We show that microglia are unable to fully restore their resting homeostatic transcriptomic state, are increased in numbers, and become activated alongside recruited immune cell populations through to 50 days post-injection (dpi). Despite evidence that microglia are activated during the inflammatory response, through pharmacological depletion we demonstrate that the initiation of GTAU is microglia independent. In contrast, genetic and pharmacologic ablation of T cells effectively prevents GTAU. When compared to corticosteroids, we show that targeting lymphocyte recruitment through sphingosine-1-phosphate receptor (S1PR) modulation can provide a steroid-sparing alternative to improve ocular gene therapy outcomes.

## RESULTS

### IVT rAAV2 administration elicits dynamic and persistent inflammatory changes in resident microglia and infiltrating populations

**Figure 1.**
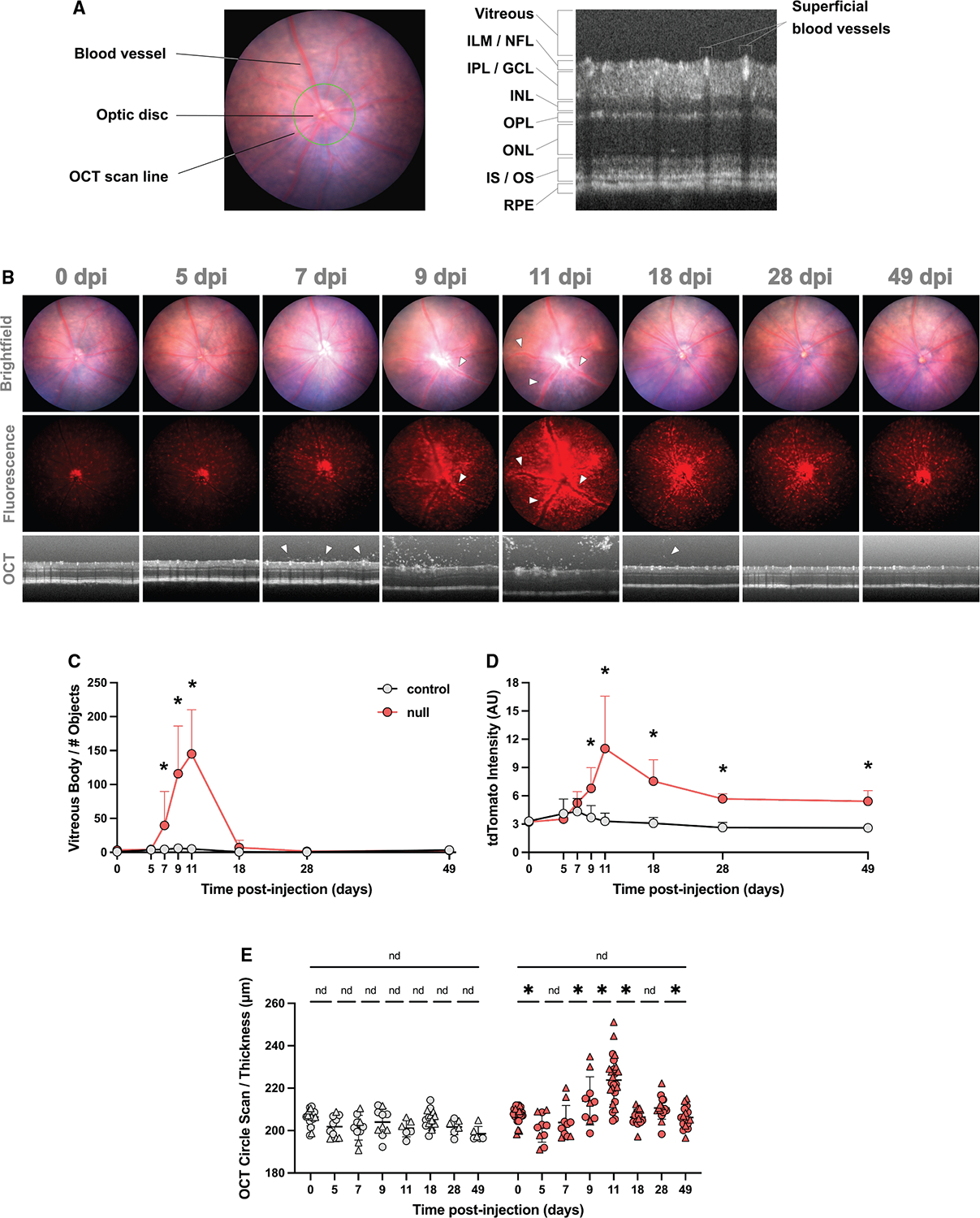
*In vivo* imaging reveals retinal microglia migrate to surround retinal vessels during intravitreal rAAV-induced GTAU *Cx3cr1*^*CreER*^*:R26-tdTomato*^+/−^ C57BL/6J mice were intravitreally injected with AAV2.CMV.null:WPRE.io2 (1E10 gc/eye) or vehicle and followed longitudinally for 49 days post-injection (dpi). (A) Representative examples of bright-field fundoscopy (left) and optical coherence tomography (OCT; right) circle scan imaging readouts, with detailed labeling of retinal layers: inner limiting membrane (ILM)/nerve fiber layer (NFL), inner plexiform later (IPL)/ganglion cell layer (GCL), inner nuclear layer (INL), outer plexiform layer (OPL), outer nuclear layer (ONL), photoreceptor inner/outer segments (IS/OS), and retinal pigment epithelium (RPE). (B) Representative images from an AAV2-injected eye with bright-field (top row), fluorescence (center row), and OCT (bottom row) imaging demonstrating the progression of rAAV-induced GTAU from peak inflammation at 11 days through clinical resolution at 49 dpi. White arrowheads indicate vasculitis/perivascular sheathing (bright field), perivascular accumulation of tdTomato^+^ retinal microglia (fluorescence), and vitritis (OCT). (C) Timeline analysis of cellular infiltration into the posterior compartment vitreous measured OCT circle scans (*n* = 6–10 per time point). (D) Timeline analysis of tdTomato brightness (mean fluorescence intensity [MFI]) (*n* = 4–6 per time point). (E) Timeline analysis of total retinal thickness measured from OCT circle scans (mixed males and females, *n* = 6–20 per time point; on graph, circles are males and triangles are females). Statistical analysis was performed by a two-way ANOVA, with multiple comparisons testing by false discovery rate (FDR) correction using the two-stage step-up method of Benjamini, Krieger, and Yekutieli. Asterisks indicate *q* ≤ 0.05, and nd indicates no discovery. Columns and error bars represent means ± SDs, respectively.

The resident population of retinal microglia can be visualized *in vivo* in C57BL/6 *Cx3cr1*^*CreER*^*:R26-tdTomato*^+/−^ mice.^37^ In these mice, we used longitudinal imaging to characterize the dynamics and kinetics of the macroscopic microglia responses following IVT administration of rAAV. Inflammation severity was assessed on fundus images obtained under bright-field or fluorescence (FS) illumination and scored quantitatively using optical coherence tomography (OCT) of the posterior segment (Figure 1A). Inflammation was assessed on days 5, 7, 9, 11, 18, 28, and 49 after IVT injection of a “null” (trans-gene-less) rAAV2 vector at 1E10 genome copies (gc) and compared to vehicle-injected controls (PBS with 0.001% Pluronic F-68 v/v).

Clinical assessment revealed that injection of AAV led to clinical vasculitis and cellular infiltration in the vitreous chamber from 7 dpi, which reached a peak at 11 dpi and appeared to resolve by 28 dpi, in line with previous reports (Figures 1B and 1C).^5,21^ Visualization of tdTomato FS at 0 dpi showed clustering of the microglia at the optic nerve, with a uniform distribution in the retina (Figure 1B). Post-injection, a gradual increase in tdTomato brightness and accumulation at the interface between superficial vasculature and the retina became detectable at 7 dpi and peaked at 12 dpi, mirroring the kinetics of cellular infiltrate. As vitritis resolved toward 28 dpi, the microglia returned to a uniform fundal distribution but notably, overall tdTomato brightness remained significantly elevated in comparison with vehicle-injected eyes to 50 dpi (Figure 1D). A transient and self-resolving retinal edema occurred concurrently with the kinetics of vitritis, but otherwise retinal structure was not impacted across the study period (Figure 1E).

**Figure 2.**
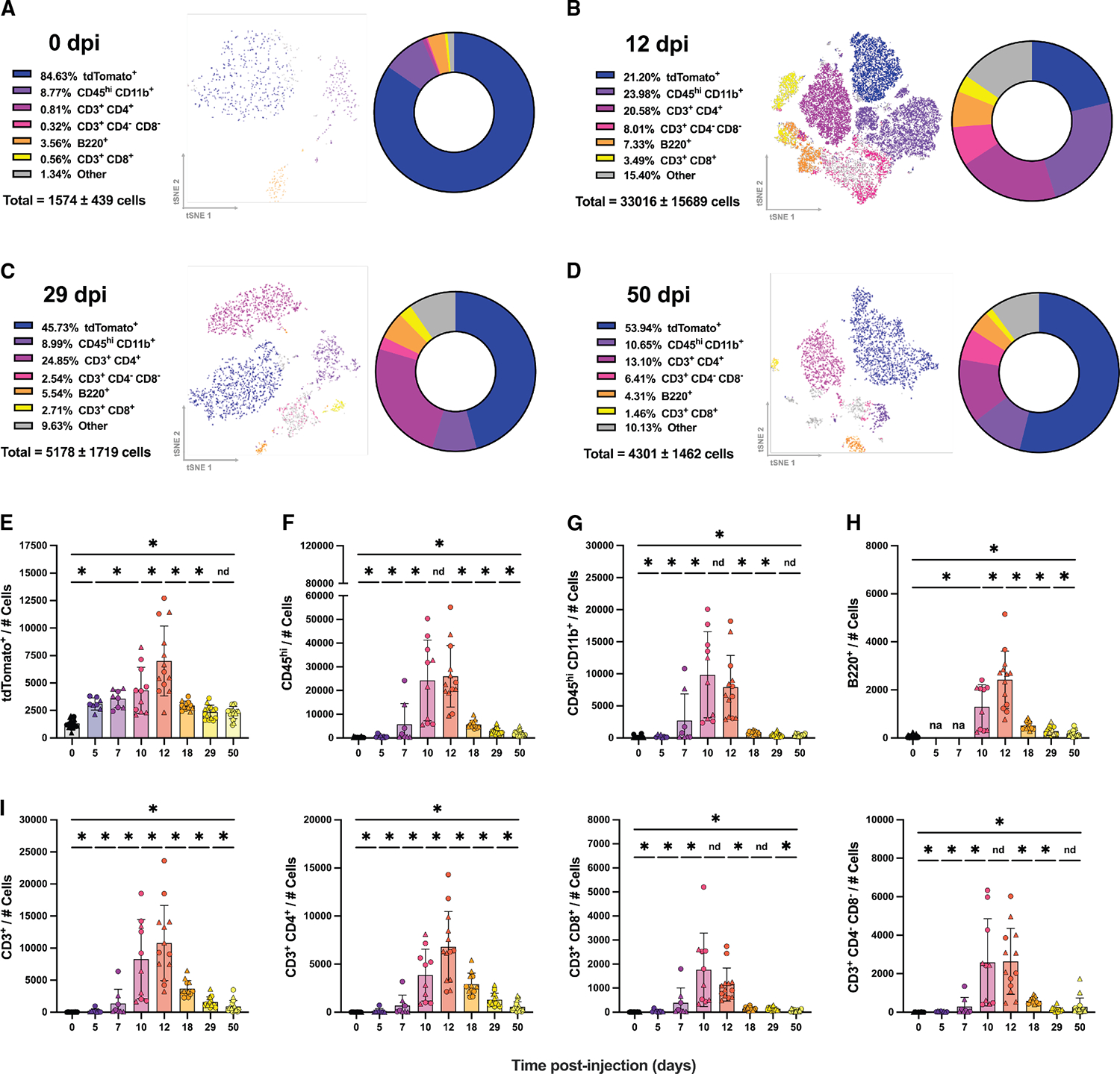
rAAV-induced GTAU significantly alters retinal immune homeostasis *Cx3cr1*^*CreER*^*:R26-tdTomato*^+/−^ C57BL/6J mice were intravitreally injected with AAV2.CMV.null:WPRE.io2 (1E10 gc/eye) or vehicle, and at select time points, CD45^+^ cells were isolated from individual retinas and analyzed for cell type by flow cytometry (full gating strategy in Figure S1). Results from multiple eyes were combined for pooled analysis. (A–D) For the time point indicated, pie graphs represent the percentage of each cell type as a percentage of total retinal CD45^+^ cells, and t-distributed stochastic neighbor embedding (tSNE) plots visualize statistical clustering of the CD45^+^ cell types. (A) Baseline (0 dpi), *n* = 10. (B) Peak inflammation (12 dpi), *n* = 13. (C) Early resolution (29 dpi), *n* = 16. (D) Late resolution/chronic phase (50 dpi), *n* = 10. The 50 dpi samples demonstrate the persistence of non-microglial (tdTomato^−^) immune cells, including CD3^+^CD4^+^ T cells (pink) when compared with baseline. (E–I) Absolute counts of resident tdTomato^+^ cells and infiltrating CD45^hi^ cell populations at 0 dpi (*n* = 10), 5 dpi (*n* = 8), 7 dpi (*n* = 8), 10 dpi (*n* = 11), 12 dpi (*n* = 13), 18 dpi (*n* = 12), 29 dpi (*n* = 16), and 50 dpi (*n* = 10) during rAAV-induced GTAU. Mixed males and females; on graphs, circles are males and triangles are females. Statistical analysis was performed by a Brown-Forsythe and Welch ANOVA, with multiple comparisons testing by FDR correction using the two-stage step-up method of Benjamini, Krieger, and Yekutieli. Asterisks indicate *q* ≤ 0.05, and nd indicates no discovery. Columns and error bars represent means ± SDs, respectively.

To define the relative contribution of retinal microglia versus infiltrating immune cell populations to GTAU, retina and vitreous from microglia reporter mice were analyzed by flow cytometry at multiple time points post-injection (Figure 2). Before injection of rAAV (0 dpi), tdTomato^+^ microglia represent the majority of CD45^+^ cells, with CD45^hi^ CD11b^+^ myeloid cells and B220^+^ cells also present at low levels (Figure 2A). During peak inflammation, at 12 dpi, overall CD45^+^ cell numbers increased 21-fold (33,016 ± 15,689 vs. 1,574 ± 439 cells), reflecting expansion of the resident tdTomato^+^ microglia as well as recruitment of CD45^hi^ peripheral leukocytes to the eye. Predominantly, these comprised CD3^+^ T cells (including CD4^+^ helper/regulatory, CD8^+^ cytotoxic, and CD4^−^ CD8^−^ double-negative subsets), followed by CD45^hi^ CD11b^+^ myeloid cells (monocytes, macrophages, and granulocytes) and B220^+^ cells (Figure 2B). Remarkably, while overall leukocyte numbers decreased nearly 8-fold from 12 to 50 dpi (4,301 ± 1,462 cells vs. 33,016 ± 15,689), both resident microglia and infiltrating populations (notably CD4^+^ T cells) remained elevated compared with 0 dpi (Figures 2C and 2D).

To further quantify the disruption of retinal immune homeostasis occurring after IVT rAAV injection, we determined the absolute number of immune cells per retina by cell type, including additional early and late time points. By 5 dpi, tdTomato^+^ cell numbers (3,095 ± 551) expanded 2-fold from 0 dpi (1,332 ± 399), increasing to 7,001 ± 3,180 (12 dpi) at peak inflammation. Notably, however, the acute expansion of the microglial population at 5 dpi was observed in both control and vector-injected animals, indicating a transitory effect of injection trauma, which receded by 12 dpi (Figure S2). As clinical inflammation subsided, microglia numbers reduced, although they remained significantly elevated at 50 dpi (2,320 ± 557) compared to 0 dpi (Figure 2E). Non-microglia CD45^hi^ infiltrating populations were detectable from 5 dpi (before initial signs of vitritis), following similar kinetics with a peak of 26,015 ± 13,010 cells at 12 dpi, and by 50 dpi decreased (1,981 ± 1,117 cells) but also remained significantly elevated compared with 0 dpi (243 ± 217 cells) (Figure 2F). The infiltrate comprised CD3^+^ populations, including CD4^+^ (T helper or regulatory cells), CD8^+^ (cytotoxic T cells), and double-negative T cells (CD3^+^ CD4^−^CD8^−^), alongside CD11b^+^ (monocytes/granulocytes) and B220^+^ B cells (Figures 2G–2I). Notably, the numbers of CD45^hi^ CD11b^+^ and CD3^+^ CD8^+^ cells reached their maximum at 10 dpi, earlier than other cell types. Critically, all leukocyte populations in the retina and vitreous, apart from CD45^hi^ CD11b^+^ cells, remained elevated at 50 dpi compared with 0 dpi, demonstrating a persistent change to the retinal immune threshold following IVT administration.

### Retinal microglia exhibit a sustained dysregulated phenotype in response to IVT rAAV administration

Having established that IVT AVV leads to dynamic changes in the number and localization of retinal microglia, we next sought to identify associated gene expression changes in known homeostatic and pro-inflammatory pathways. Furthermore, to test the hypothesis that retinal microglia function as key immune cells in the eye, we first sought evidence for innate inflammatory pathway activation following AAV exposure. This analysis utilized our previously published microglia mRNA sequencing (mRNA-seq) dataset generated from the retinas of microglia reporter mice exposed to IVT AAV (GSE266332; Figure S3 for analysis strategy).^20^

**Figure 3.**
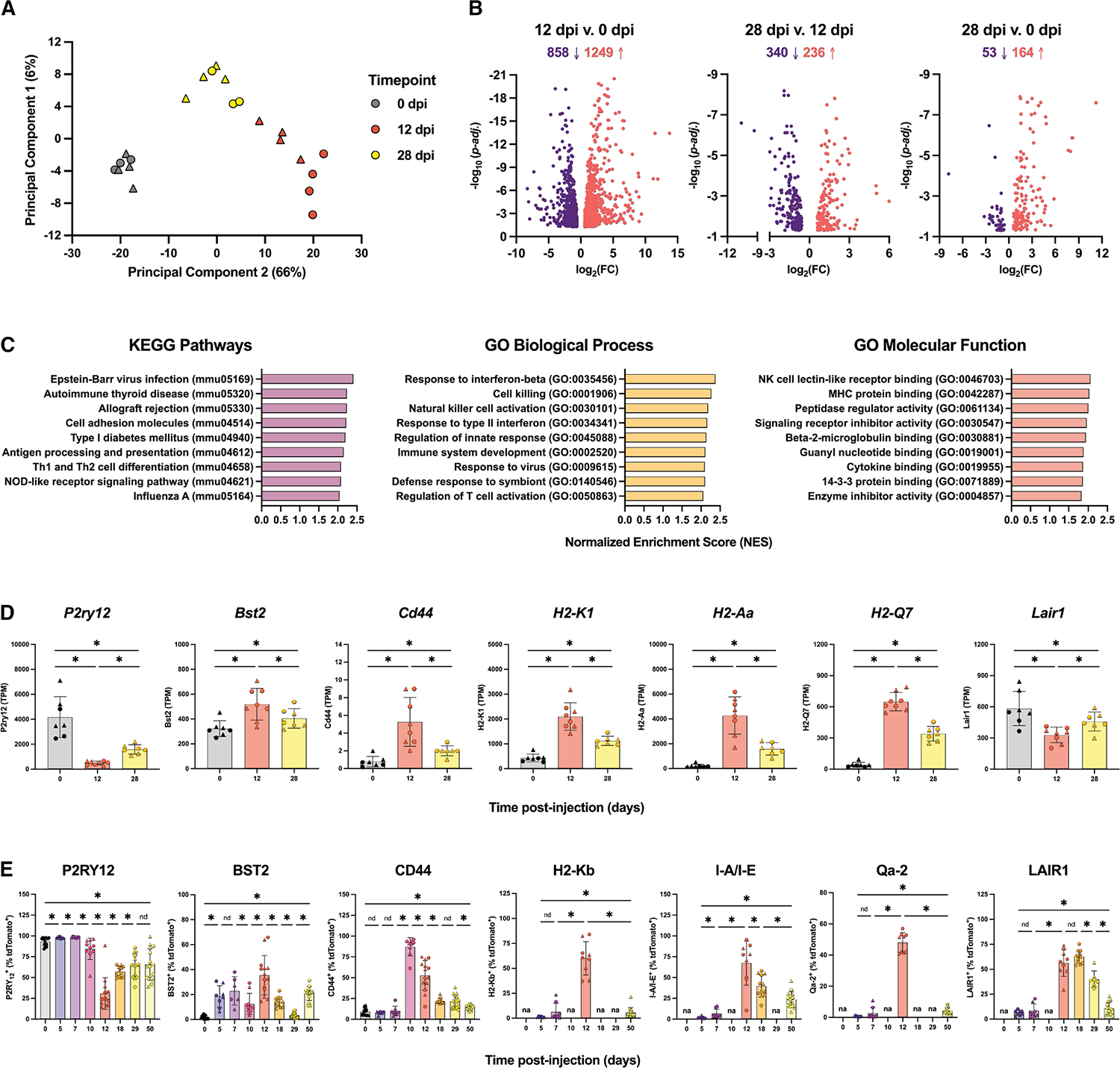
Retinal microglia remodel their transcriptome and proteome in response to rAAV *Cx3cr1*^*CreER*^*:R26-tdTomato*^+/−^ C57BL/6J microglia reporter mice were intravitreally injected with AAV2.CMV.null:WPRE.io2 (1E10 gc/eye) at 0 dpi and analyzed by bulk RNA sequencing or flow cytometry. Retinas were harvested on 0 dpi (*n* = 7), 12 dpi (*n* = 8), and 28 dpi (*n* = 7), and 600 tdTomato^+^ microglia were sequenced to a depth of 6.0 million reads. (A) Principal-component analysis plot of transcriptomic changes during the first 28 days of rAAV-induced GTAU. (B) Visualization and quantification of upregulated and downregulated genes between samples harvested at 0, 12, and 28 dpi based on analysis by DESeq2. (C) Functional gene set enrichment analysis of 12-dpi DEGs according to Kyoto Encyclopedia of Genes and Genomes (KEGG) pathways and non-redundant Gene Ontology (GO) terms. (D) Comparison of TPM (transcripts per kilobase million) of selected microglial homeostasis (*P2ry12*) and activation (*Bst2*, *Cd44*, *H2–K1*, *H2-Aa*, *H2–Q7*, and *Lair1*) markers at 0, 12, and 28 dpi. (E) Flow analysis for co-expression of selected microglial homeostasis (P2RY12) and activation proteins (BST2, CD44, H2-Kb, I-A/I-E, Qa-2, and LAIR1) with CD45 on single tdTomato^+^ retinal cells was measured at 0, 5, 7, 10, 12, 18, 29, and 50 dpi (*n* = 7–16 per time point). Animal sex is indicated in (A), (D), and (E) with circles for males and triangles for females. na indicates lack of data at selected time points. Statistical analysis was performed by a Brown-Forsythe and Welch ANOVA, with multiple comparisons testing by FDR correction using the two-stage step-up method of Benjamini, Krieger, and Yekutieli. Asterisks indicate *q* ≤ 0.05, and nd indicates no discovery. Columns and error bars represent means ± SDs, respectively.

Principal-component analysis provided clear separation of 0 dpi from the AAV-exposed samples (12 and 28 dpi) and moderate separation according to the post-injection time point (Figure 3A). The difference between samples from 0 to 28 dpi was less pronounced but distinctly two-dimensional, indicating an incomplete recovery toward homeostasis. Analysis of differentially expressed genes (DEGs) confirmed the greatest transcriptomic shift between samples from naive and 12-dpi animals (2,107 DEGs), followed by 12–28 dpi (576 DEGs) and 0–28 dpi (217 DEGs) (Figure 3B; Tables S1, S2, S3, S4, S5, and S6).

Next, we performed *in silico* pathway analyses aimed at identifying canonical pathways, upstream regulators, and causal networks activated in retinal microglia in response to AAV exposure. During the peak response (12 dpi), microglia had enrichment of genes associated with interferon (IFN)-β and IFN-γ-driven antiviral responses, cell proliferation, migration, adhesion, and antigen processing and presentation, as well as signatures of infection by a virus (Figures 3C and S4; Tables S7, S8, S9, S10, S11, S12, S13, S14, S15, S16, S17, and S18). However, when compared with naive microglia, 28 dpi remained broadly pro-inflammatory, with enrichment of antigen presentation to T cell receptors/B cell receptors, IFN, and interleukin (IL)-1/4/8 signaling, and phagosome formation upregulated by predicted upstream activity of STAT1/5B, IRF1/3, and TRIM26 (Tables S19, S20, S21, S22, S23, S24, S25, and S26).

Key microglial genes associated with homeostasis,^38^ activation,^39,40^ antigen presentation,^41^ and suppression^42–45^ (Figure 3D; Table S27) were evaluated at the protein level.^36^ Importantly, extended analysis of these markers at additional time points demonstrated that despite gradual normalization from peak inflammation (12 dpi), expression remained persistently dysregulated at 50 dpi (Figure 3E). Collectively, the data demonstrate that AAV exposure significantly alters retinal microglial, transcription, and protein expression in a manner consistent with initiation of the innate immune response to AAV. These data also suggest that retinal microglia may participate in the chronic manifestations of GTAU through increased expression of genes such as major histocompatibility complex class II (MHC class II) that are important in coordinating adaptive immune responses.

### Microglia-independent mechanisms drive the induction and resolution of GTAU

**Figure 4.**
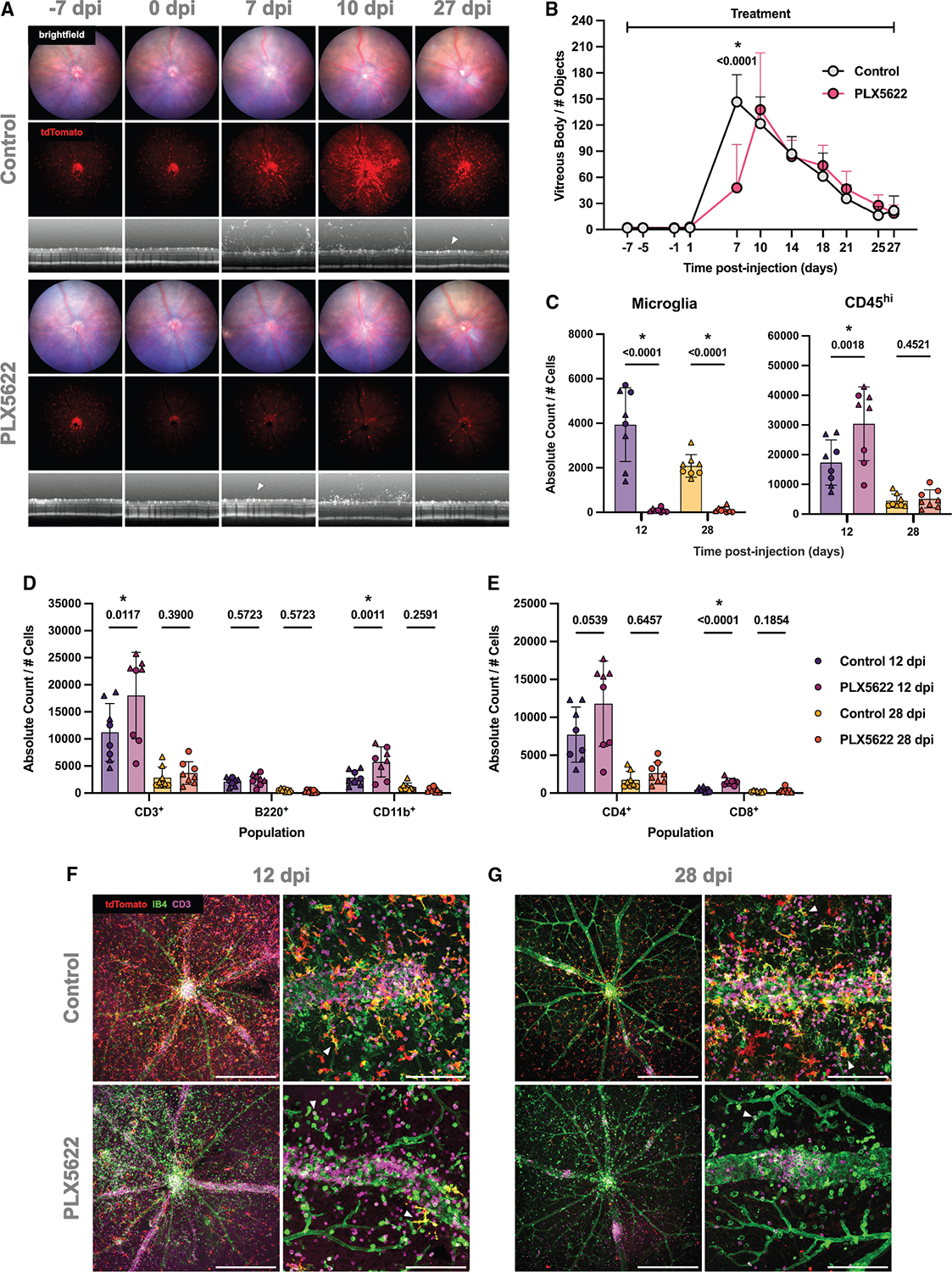
Retinal microglia are not essential for the initiation of rAAV-induced GTAU *Cx3cr1*^*CreER*^*:R26-tdTomato*^+/−^ C57BL/6J microglial reporter mice were treated with a diet containing PLX5622 (1,200 ppm) or control chow beginning at −7 dpi, and AAV2.CMV.null:WPRE.io2 (1E10 gc/eye) was intravitreally injected at 0 dpi. Retina and vitreous from single eyes processed for flow cytometric analysis at 12 dpi (*n* = 8) and 28 dpi (*n* = 8). Mouse sex indicated on graphs in (C)–(E); circles are males and triangles are females. (A) Representative fundus bright-field, tdTomato fluorescence, and OCT images from control and PLX5622-treated animals on −7, 0, 10, and 27 dpi showing progression of rAAV-induced GTAU. tdTomato fluorescence is notably decreased in the PLX5622-treated animals. (B) Timeline analysis of cellular infiltration into the vitreous body, measured by ImageJ/Fiji from *in vivo* Micron IV OCT circle scans (*n* = 5–16 per time point). (C) Absolute counts of tdTomato^+^ microglia and infiltrating CD45^hi^ cells at 12 and 28 dpi. (D) Absolute counts of specific CD45^hi^ populations, including CD3^+^, B220^+^, and CD11b^+^ cells, at 12 and 28 dpi. (E) Absolute counts of CD3^+^ (CD4^+^ and CD8^+^) subsets at 12 and 28 dpi. (F and G) Representative retinal flatmount immunofluorescence images show CD3^+^ (magenta) cells, tdTomato^+^ microglia (red), and isolectin B4 (IB4) binding (green) at 12 and 28 dpi. Each condition and time point comprises images at 10× (scale bars, 500 μm) and 40× (scale bars, 125 μm) magnification. Statistical analysis was performed by two-way ANOVA or factorial aligned rank transform ANOVA, with multiple comparisons testing by FDR correction using the two-stage step-up method of Benjamini, Krieger, and Yekutieli or the original method of Benjamini and Hochberg, respectively. *q ≤ 0.05, and nd indicates no discovery.

To test the requirement for retinal microglia in the initiation of GTAU, PLX5622 (CSFR1 antagonist) was used to deplete microglia prior to IVT rAAV injection in microglia reporter mice. Surprisingly, GTAU course and severity was not abrogated by PLX5622 treatment (Figures 4A and 4B). Instead, PLX5622 treatment significantly increased the number of ocular CD45^hi^ cells, including CD3^+^ and CD45^hi^ CD11b^+^ populations (Figures 4C–4E). Successful depletion of microglia was confirmed by the absence of tdTomato fundus FS at peak inflammation in PLX5622-treated animals (Figures 4A and 4C) and on immunohistochemistry (IHC) of retinal flatmounts on 12 dpi (Figure 4F). At 28 dpi, no differences were observed between the PLX5622-treated and control-treated animals in total numbers of retinal CD45^hi^ cells or their subsets (which remained elevated compared to naive). Representative *ex vivo* retinal flatmounts from 28 dpi demonstrate the sustained depletion of microglia in PLX5622-treated animals and the ability of IB4^+^ tdTomato^−^ monocytes and CD3^+^ T cells to extravasate independently of the microglia (Figure 4G). These findings demonstrate that retinal microglia are not necessary for the initiation and resolution of GTAU and suggest a regulatory rather than pathogenic role in the early response.

### Inhibition of lymphocyte recruitment prevents rAAV-induced GTAU and dysregulated microglial phenotype

**Figure 5.**
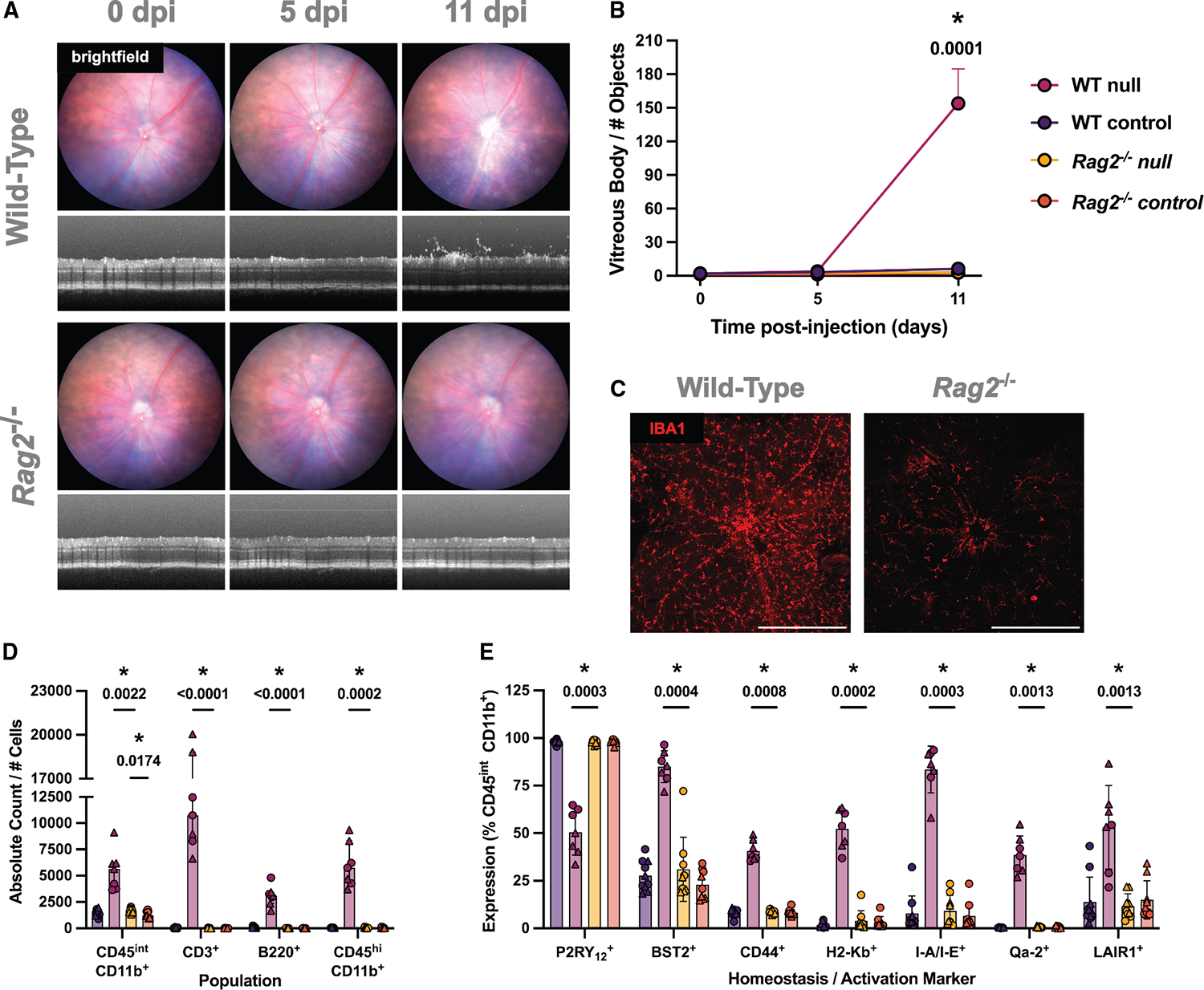
Lymphocytes are necessary for rAAV-induced GTAU C57BL/6J (wild-type) and B6.Cg-Thy1 (*Rag2* knockout) mice were intravitreally injected with AAV2.CMV.null:WPRE.io2 (1E10 gc/eye) or vehicle at 0 dpi. Retina and vitreous from single eyes were processed into single-cell suspensions for flow cytometric analysis at 12 dpi. Alternatively, retinal flatmounts were prepared for confocal fluorescence microscopy. (A) Representative clinical and subclinical images (fundus bright-field, tdTomato fluorescence, OCT circle scan) captured at 0, 5, and 11 dpi. (B) Timeline analysis of cellular infiltration into the vitreous body from OCT circle scans (*n* = 7–10). (C) Images of *ex vivo* flatmount immunofluorescence staining for IBA1^+^ cells (microglia/macrophages) of rAAV-injected wild-type and *Rag2* knockout eyes (scale bars, 500 μm). (D) Absolute counts of microglia (CD45^int^ CD11b^+^; approximate) and infiltrating CD45^hi^ cells in the retina and vitreous (*n* = 7–10). (E) Protein expression of selected microglial homeostasis (P2RY12) and activation (BST2, CD44, H2-Kb, I-A/I-E, Qa-2, and LAIR1) markers on CD45^mid^ CD11b^+^ cells (*n* = 7–10). Statistical analysis was performed by two-way ANOVA, with multiple comparisons testing by FDR correction using the two-stage step-up method of Benjamini, Krieger, and Yekutieli. Asterisks indicate *q* ≤ 0.05, and nd indicates no discovery. Columns and error bars represent means ± SDs, respectively.

Recognizing the predominance of CD3^+^ T cells in GTAU and potential role of posterior ocular lymphatic drainage,^24^ we tested the hypothesis that ocular infiltrating lymphocytes are necessary for the GTAU response, using a genetic approach to inhibit their recruitment. Wild-type (WT) C57BL/6J and B6.Cg-Thy1 (*Rag2* knockout) mice, in which the development of mature T and B cells is abolished, received rAAV or control injections with clinical monitoring until 12 dpi. Compared with WT, RAG2-deficient animals lacked all signs of clinical inflammation (vasculitis, perivascular sheathing, vitritis). This was confirmed by IHC, with no retinal inflammation evident in *Rag2* knockout animals and absence of IBA1^+^ cells at the retinal vasculature, which was prominent in WT mice (Figures 5A–5C). Flow cytometry identified significantly fewer CD45^+^ cells (CD45^hi^, CD3^+^, B220^+^, or CD11b^+^) at 12 dpi in RAG2-deficient retinas when compared to WT counterparts (Figures 5D and S5 for gating strategy). Furthermore, microglia cell (CD45^int^ CD11b^+^) numbers were not significantly increased above baseline in *RAG* retinas (1,688 ± 237 vs. 1,343 ± 296 cells), with no alteration in protein expression of homeostatic and proinflammatory markers (Figures 5D and 5E).

### Immunomodulatory strategies to suppress lymphocyte infiltrate are superior for GTAU inhibition

**Figure 6.**
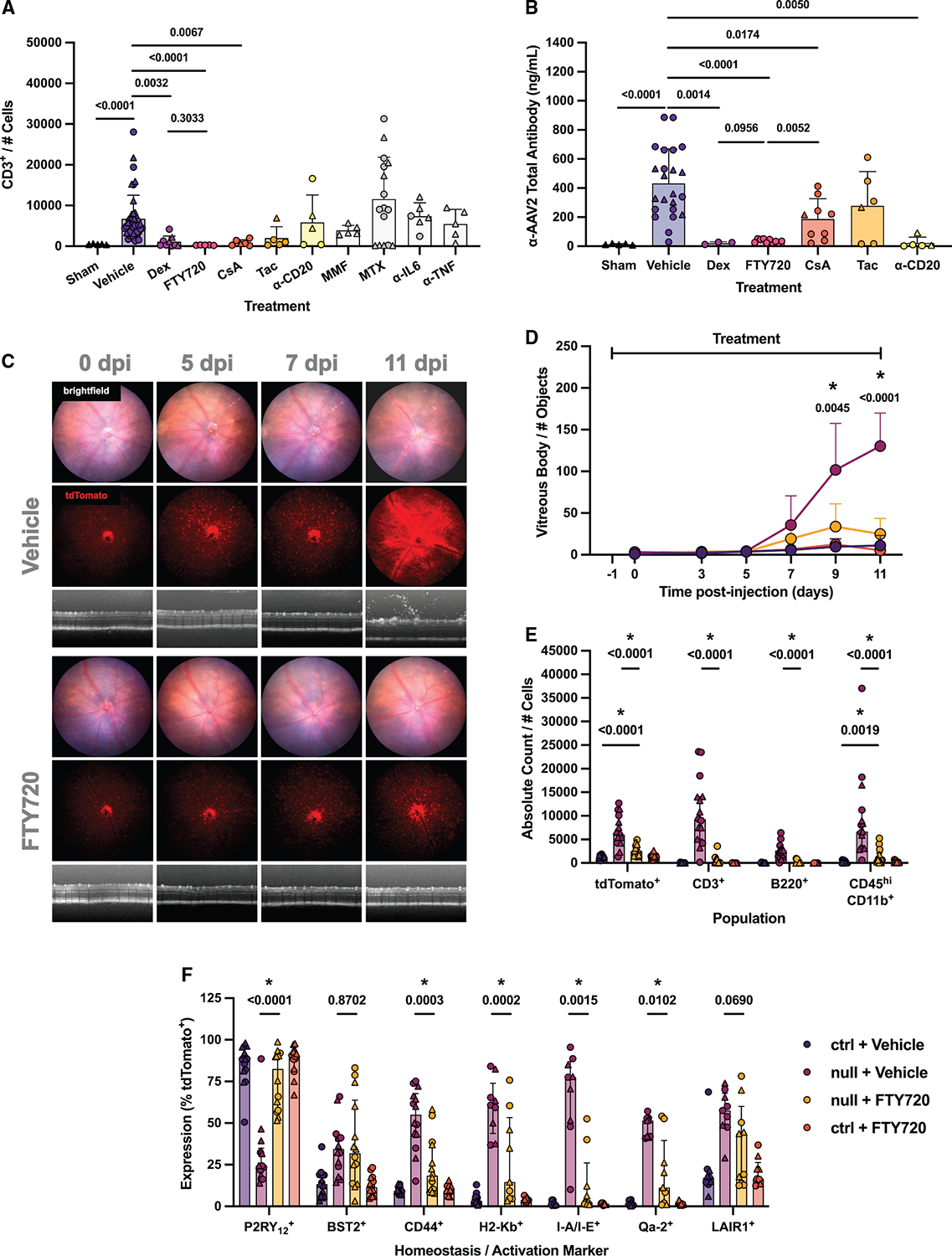
Evaluation of pharmacologic strategies reveals potential of sphingosine-1-phosphate modulation to prevent rAAV-induced GTAU Wild-type C57BL/6J mice receiving AAV2.CMV.eGFP:WPRE (5.4E9 gc/eye) or sham (PBS) at 0 dpi were treated with fingolimod (FTY720), dexamethasone (Dex), methotrexate (MTX), mycophenolate mofetil (MMF), cyclosporine A (CsA), tacrolimus (Tac), and mouse-specific neutralizing antibodies to tumor necrosis factor α (TNF-α), IL-6, and CD20 or vehicle (PBS) until 28 dpi (specific dosing regimens and administration routes provided in materials and methods). Whole single eyes were processed for flow cytometric analysis and serum assayed for anti-AAV2 capsid-binding antibodies at 28 dpi. (A) Absolute counts of CD3^+^ cells. (B) Titers of anti-AAV2 capsid-binding serum antibodies at 28 dpi (*n* = 3–5 per treatment group, *n* = 23 for vehicle). *Cx3cr1*^*CreER*^*:R26-tdTomato*^+/−^ C57BL/6J microglia reporter mice received AAV2.CMV.null:WPRE.io2 (1E10 gc/eye) or control and treated with FTY720 (6 mg/kg) or vehicle administered by oral gavage every other day from −1 to 11 dpi. Retina and vitreous from single eyes were prepared for flow cytometric analysis at 12 dpi. (C) Representative clinical and subclinical images (fundus bright-field, tdTomato fluorescence, OCT circle scan) captured at 0, 5, 7, and 11 dpi. (D) Timeline analysis of cellular infiltration into the vitreous body, measured from OCT circle scans (*n* = 9–15). (E) Absolute counts of tdTomato^+^ microglia and infiltrating CD45^hi^ cells, including CD3^+^, B220^+^, and CD11b^+^ populations (*n* = 9–15). (F) Protein expression of selected microglial homeostasis (P2RY12) and activation (BST2, CD44, H2-Kb, I-A/I-E, Qa-2, and LAIR1) markers on tdTomato^+^ microglia (*n* = 9–15). Statistical analysis was performed by two-way ANOVA or mixed-effects analysis, with multiple comparisons testing by FDR correction using the two-stage step-up method of Benjamini, Krieger, and Yekutieli. Asterisks indicate *q* ≤ 0.05, and nd indicates no discovery. Columns and error bars represent means ± SDs, respectively.

Having highlighted the key role for lymphocytes in driving GTAU, we conducted rational screening of available immunomodulatory strategies, including lymphocyte-targeting compounds, for their efficacy in preventing rAAV-induced GTAU. Applying a prophylactic repeated dosing regimen in WT mice until 28 dpi, we screened clinically relevant and approved agents, including dexamethasone (Dex),^46^ fingolimod (FTY720),^47^ cyclosporine A (CsA),^48^ tacrolimus (Tac),^48^ methotrexate (MTX),^49^ mycophenolate mofetil (MMF),^50^ fenofibrate (Fen),^51^ pioglitazone (Pio),^52^ and mouse-specific neutralizing antibodies against tumor necrosis factor alpha (TNF-α),^53^ IL-6,^54^ and CD20.^55^ This approach identified that Dex, FTY720, and CsA were effective at reducing the absolute number of ocular infiltrating CD3^+^ cells and serum concentration of α-AAV2 total antibodies (tAb) through 28 dpi (Figures 6A, 6B, S6A, S6B, and S7 for gating strategy). Critically, however, FTY720 treatment produced 5-fold lower α-AAV2 tAb titers than CsA administration (36.21 ± 12.08 vs. 187.1 ± 139.6 ng/mL), emerging as the most effective steroid-sparing approach tested. None of the agents impacted the number of EGFP^+^ transduced cells or α-EGFP antibody titers (Figures S6C and S6D). Notably, while treatment with α-CD20 antibodies did not prevent ocular manifestation of GTAU, serum α-AAV2 tAb concentrations were significantly decreased.

FTY720 is a S1PR modulator that inhibits T cell egress and migration from lymph nodes to sites of inflammation.^56,57^ To further explore the therapeutic utility of FTY720, we assessed the effect of short-term repeated treatment to modulate the primary peak of GTAU inflammation and microglia phenotype using tdTomato reporter mice. Clinical imaging demonstrates animals receiving FTY720 exhibited no clinical or subclinical inflammation, including vitritis, retinal edema, or changes in the uniform fundal distribution of tdTomato^+^ microglia by 12 dpi (Figures 6C, 6D, and S6G). Flow cytometry confirmed the efficacy of FTY720 treatment, with inhibited CD45^hi^ (CD3^+^, B220^+^, or CD11b^+^) cell infiltration at 12 dpi (Figures 6E and S6H). Furthermore, FTY720-treated animals contained 61% fewer retinal microglia compared with vehicle treatment (2,797 ± 1,231 vs. 7,221 ± 3,164 cells), maintained normal expression of P2RY12, and exhibited limited upregulation of activation markers (Figure 6F). These data support the pharmacologic blockade of lymphocyte recruitment through S1PR modulation as an effective strategy to prevent peak cellular inflammation and activation of microglia at 12 dpi.

To evaluate whether short-term treatment with FTY720 (through the period of peak inflammation) is sufficient to prevent long-term manifestations of GTAU, FTY720 was administered for either 11 or 25 dpi, and following withdrawal, inflammation was assessed on 18 dpi or 50 dpi, respectively. Treatment through 11 dpi only delayed the onset of peak inflammation, with clinical signs (vitritis and perivascular accumulation of microglia) developing 4 days after treatment ended (on 15 dpi). Flow cytometric analysis at 18 dpi confirmed significant increases in CD45^+^ cellular infiltrate and pro-inflammatory changes to microglial phenotype (Figures S8A–S8D).

**Figure 7.**
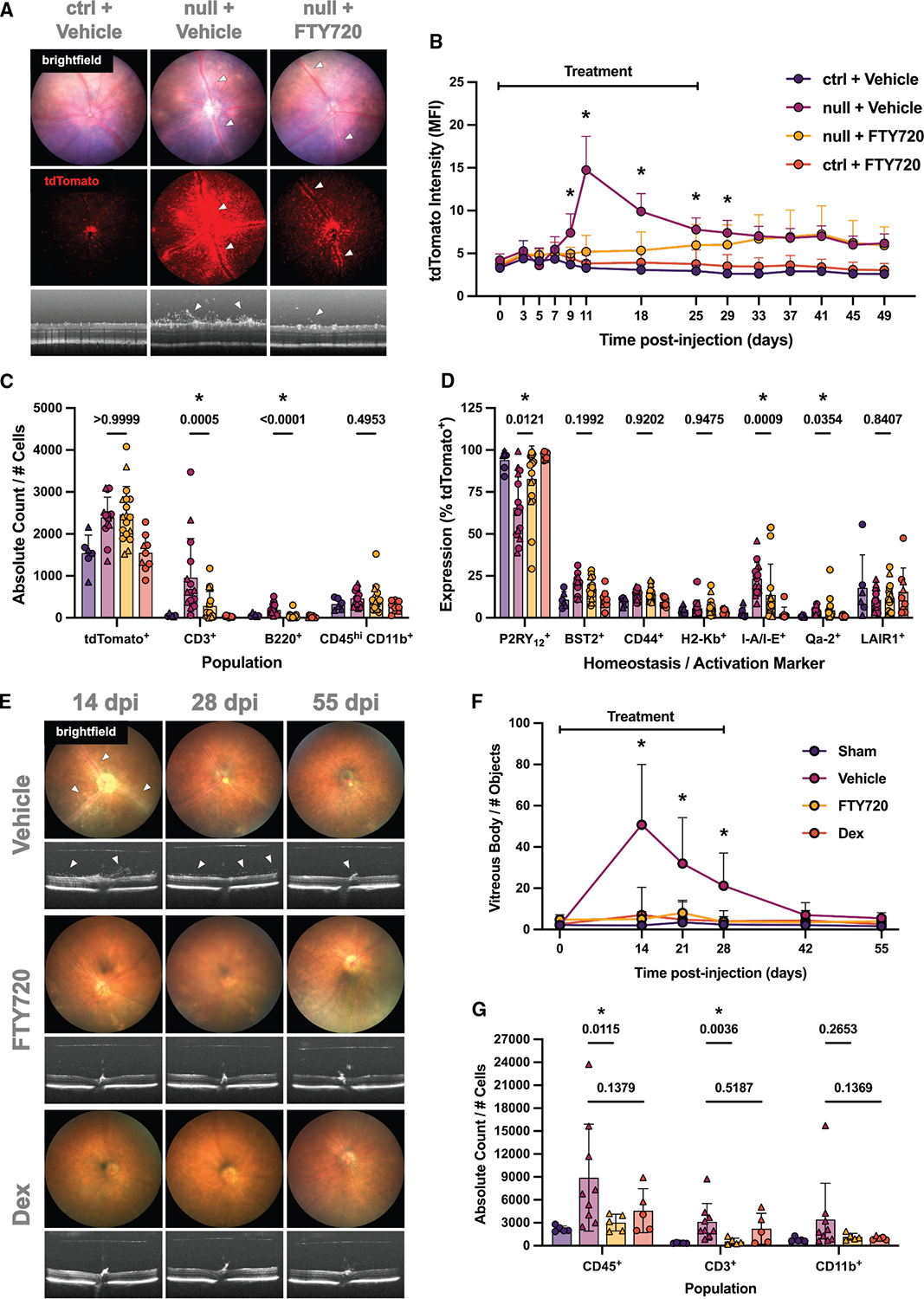
Short-term sphingosine-1-phosphate modulation produces long-term amelioration of rAAV-induced GTAU, equivalent to glucocorticoid treatment *Cx3cr1*^*CreER*^*:R26-tdTomato*^+/−^ C57BL/6J microglial reporter mice receiving AAV2.CMV.null:WPRE.io2 (1E10 gc/eye) or control were treated with FTY720 (6 mg/kg) or vehicle administered by oral gavage every other day from −1 to 25 dpi. Following withdrawal of treatment, mice were clinically monitored until 50 dpi and single eyes (retina and vitreous) processed for flow cytometry. (A) Comparison of representative peak inflammatory phenotypes from *in vivo* images. White arrowheads indicate vasculitis/perivascular sheathing (fundus) and vitritis (OCT). (B) Timeline analysis of tdTomato brightness (MFI) (*n* = 9–15). (C) Absolute counts of tdTomato^+^ microglia and infiltrating CD45^hi^ cells, including CD3^+^, B220^+^, and CD11b^+^ populations (*n* = 9–15). (D) Protein expression of selected microglial homeostasis (P2RY12) and activation (BST2, CD44, H2-Kb, I-A/I-E, Qa-2, and LAIR1) markers on tdTomato^+^ microglia (*n* = 9–15). To compare the effectiveness of S1PR modulation versus glucocorticoids, wild-type C57BL/6J mice receiving AAV2.CMV.eGFP:WPRE (5.4E9 gc/eye) or sham (PBS) were treated with vehicle (PBS), FTY720 (3 mg/kg), or high-dose Dex (2 mg/kg) tapered by decreasing the dose by half each successive week (Dex −1 to 7 dpi, 2 mg/kg; 8–14 dpi, 1 mg/kg; 15–21 dpi, 0.5 mg/kg; 22–28 dpi, 0.25 mg/kg) administered intraperitoneally daily from −1 to 28 dpi. Mice were clinically monitored until 56 dpi and whole eyes (posterior and anterior compartments) processed for flow cytometric analysis. (E) Representative clinical images (fundus bright-field and OCT line scan) captured on 12, 28, and 56 dpi. (F) Timeline analysis of cellular infiltration into the vitreous body, measured from OCT line scans. (G) Absolute counts of CD45^+^, CD3^+^, and CD11b^+^ cells at 56 dpi (*n* = 4–5/group). Statistical analysis was performed by a Shapiro-Wilk test, followed by a parametric two-way ANOVA or non-parametric factorial aligned rank transform ANOVA, with multiple comparisons testing by FDR correction using the two-stage step-up method of Benjamini, Krieger, and Yekutieli or the original method of Benjamini and Hochberg, respectively. Data reported as individual values, with columns and error bars indicating means ± SDs. Asterisks indicate *q* ≤ 0.05, and nd indicates no discovery.

In contrast, extended therapeutic treatment with FTY720 until 25 dpi demonstrated evidence of prolonged suppression of inflammation at 50 dpi (Figure 7A). During the withdrawal period, 50% (12/24) of eyes examined showed no evidence or minimal extent of clinical inflammation (vitritis) and microglial activation (elevated FS or altered distribution) (Figure S9A). In 25% (6/24) of eyes, moderate subclinical responses with limited vitritis and focal perivascular accumulation of tdTomato^+^ cells were observed 6–20 days post-withdrawal (dpw) (Figure S9B). In 4% (1/24) of eyes, typical GTAU with characteristic vitritis and widespread perivascular accumulation of tdTomato^+^ cells developed at 20 dpw (Figure S9C). Notably, in 21% (5/24) of eyes, immune responses of varying intensity developed during the treatment period to 25 dpi despite FTY720 administration (Figure S9D).

Quibit a significant decrease compared with vehicle treatment during peak inflammation (9–29 dpi) (Figure 7B). However, by 33 dpi (after clinical disease resolved spontaneously in untreated animals), tdTomato expression in both groups equalized and remained equivalent but elevated over control-injected eyes throughout the remainder of the withdrawal period.

Flow cytometric analysis at 50 dpi demonstrated that FTY720-treated eyes contained 70% fewer CD3^+^ (384 ± 345 vs. 959 ± 931) cells and 82% fewer B220^+^ (49 ± 67 vs. 189 ± 134) cells (Figure 7C). Furthermore, while the numbers of microglia at 50 dpi were equivalent between the eyes of FTY720 and vehicle-treated animals, their functional phenotype was partly ameliorated, as evidenced by 26% higher P2RY12 (82.81% ± 19.55% vs. 65.65% ± 19.04%), 21% lower I-A/I-E (MHC class II; 13.68% ± 18.32% vs. 23.31% ± 9.687%), and 25% lower Qa-2 (MHC class I; 2.62% ± 3.53% vs. 3.50% ± 1.95%) expression compared with vehicle-treated counterparts (Figure 7D).

### S1PR modulation demonstrates equivalent efficacy to glucocorticoid receptor-α agonism

Finally, to evaluate the effectiveness of S1PR modulation compared to glucocorticoids (current clinical standard), WT C57BL/6J mice receiving IVT rAAV were treated daily with FTY720 or Dex until 28 dpi and monitored following treatment withdrawal until 56 dpi. Clinical imaging revealed suppression of retinal vascular inflammation and significant decreases in the number of vitreous opacities quantified by OCT on 14, 21, and 28 dpi in both the FTY720- and Dex-treated groups, with effects persisting after treatment withdrawal through 56 dpi (Figures 7E and 7F). Flow cytometric analysis of whole eyes (combined posterior and anterior compartments) at 56 dpi confirmed an overall reduction in cellular infiltrate following treatment, with animals receiving FTY720 but not Dex demonstrating significantly fewer CD45^+^ and CD3^+^ counts when compared with untreated controls (Figure 7G). Serum concentrations of α-AAV2 tAb from FTY720 or Dex at 56 dpi were not significantly different compared with the untreated controls following withdrawal (Figure S6E).

## DISCUSSION

The inflammatory response elicited to rAAV vectors continues to present a critical challenge for the long-term efficacy and safety of gene therapy in the eye. Here, we extend our previous mechanistic studies in GTAU^20^ to demonstrate that IVT administration of rAAV2 leads to a persistent and dysregulated ocular immune environment. Surprisingly, our findings reveal that retinal microglia are not necessary for the initiation of GTAU. In contrast, adaptive immune cells, specifically T cells, are necessary for both acute and chronic inflammation. Importantly, we provide novel preclinical data that GTAU can be prevented by blocking T cell migration with short-term S1PR inhibition. These data bring new insight regarding the contributions of resident and peripheral immune cells in shaping rAAV-mediated inflammation and emphasize the importance of building our understanding of the inflammatory processes in GTAU, to better tailor management strategies for ocular gene therapy.

Previous work from our group and others has shown that *in vivo* imaging confirms IVT delivery of rAAV drives a robust peak of inflammation (with clinical signs of perivascular sheathing and vitritis) around 12 dpi, which spontaneously resolves in the majority of eyes by 28 dpi.^5,20,21^ Here, we extend these observations until 50 dpi and use the *Cx3cr1*^*CreER*^*:R26-tdTomato*^+/−^ reporter mouse strain to reveal new features of the retina resident immune cell responses to rAAV. Previous bright-field imaging identified perivascular accumulations of immune cells, described clinically as “sheathing.” Using the tdTomato reporter line, we now understand that retinal microglia expand in number and migrate to participate in this feature of acute inflammation. While microglia resume a uniform fundal distribution, elevated tdTomato expression until 50 dpi indicated that rAAV elicits long-term changes in the resident immune compartment (Figure 1). Using established markers of microglial activation,^36^ flow cytometric analysis demonstrates an increased number of microglia exhibiting an activated phenotype (CD44, BST2, LAIR1), with concomitant reduction of homeostatic P2RY12 at peak. As inflammation subsides, P2RY12 expression was only partially restored, with elevated numbers of microglia and recruited CD45^+^ (innate and adaptive) populations remaining until 50 dpi (Figure 2). This extends the concept that rAAV2 injection leads to permanent changes in the tissue and reprogramming of the normal immunosurveillance mechanisms of the eye.^58^

Analysis of single-eye mRNA-seq data further highlights that significant inflammation-responsive transcriptional changes at 12 dpi remain altered at 28 dpi, indicating that microglia are unable to fully reset or restore their resting homeostatic phenotype (Figures 3 and S4). At peak (12 dpi), the molecular signature is characterized by the downregulation of canonical homeostatic genes, including *P2ry12*, *Tmem119*, and *Siglech*, which remain perturbed compared with the naive state. Increased expression of *Cst7*, *Apoe*, *Axl*, *Itgax*, *Lgals3*, and *Spp1* suggests that rAAV may promote damage-associated microglia-like or microglial neurodegenerative-like phenotypes that persist following the resolution of clinical inflammation at 28 dpi (Figure S4G). In the eye, dysregulation of homeostatic microglia signature genes is associated with photoreceptor degeneration.^29^ However, as we found no clinical evidence of retinal thinning or pathology by 50 dpi, an extended assessment (6–12 months) would be required to determine whether such transcriptomic changes are linked and contribute to a degenerative phenotype. Increasing evidence shows a positive correlation between vector dose and choroidal atrophy (CRA) severity following subretinal rAAV administration, and while the exact etiology of CRA remains uncertain, some features suggest an immunologic or inflammatory origin.^59,60^ Additionally, our recent work showed that an enhanced dysregulated microglia phenotype at 28 dpi was linked to tissue degeneration in aged female mice, highlighting the risk that this change in signature can pose.^20^

Notwithstanding, microglia exhibit an enhanced response and upregulation of viral-specific transcripts associated with an IFN response (*Irf7* and *Irf1)*, recognition of viral proteins (*Tlr1*, *Tlr2*), and cell proliferation (*Dna2*, *Psrc1*, *Haus8*, *Id2*, and *Pim1*) but also genes involved with antigen presentation (*H2–K1*, MHC class I; *H2-Aa*, MHC class II; and *H2–Q7*, Qa-2) pathways (Figure S4). Using flow cytometry, we demonstrate that as the inflammatory response develops, microglia upregulate the expression of MHC classes I and II at peak clinical inflammation, which reduces but remains elevated compared to 0 dpi by 50 dpi. This implies that microglia have a functional role to present AAV antigens and amplify the GTAU response but also retain a long-term capacity to present both intracellular and extracellular AAV antigens to CD8^+^ cytotoxic and CD4^+^ T helper cells. Similar expression kinetics of Qa-2 (a non-classical MHC class I) further raises the potential that microglia may also modulate/influence the GTAU response through interaction with CD8^+^ and NK cells, to promote non-classical CD8 responses as seen in chronic viral infections.^61^ Coupled to the observed perivascular accumulation of microglia (at peak inflammation) and long-term proximity to CD^3+^ cells at the vascular interface (evident at 28 dpi; Figure 4E), implicates this functional phenotype in the progression of GTAU. In support, non-human primate (NHP) tissue sections following subretinal rAAV8 delivery demonstrate evidence of microglial activation and MHC class I and class II expression in the presence of CD8^+^ and B cells at peak inflammation.^23^

Under normal conditions, retinal microglia do not express MHC class II and only when activated are considered to have antigen-presenting capacity.^62–64^ Evidence from preclinical models of autoimmune uveitis (EAU) highlights that microglia increase the expression of MHC class II during the amplification and peak stages of inflammation, and their critical role in the initiation of disease during the early stages occurs via antigen-nonspecific mechanisms.^25,26^ However, in the context of GTAU, application of the CSF1R antagonist PLX5622 revealed no change to the overall cellular kinetics in terms of onset, peak, and resolution in the absence of microglia (Figure 4). Instead, the increased frequency of CD45^hi^ infiltrate (both CD3^+^ and CD11b^+^) at peak time points may reflect an attempt by retinal microglia to regulate and protect the tissue from excessive inflammation. Nonetheless, the inflammatory response remains self-limiting, with comparable clinical and subclinical outcomes at 28 dpi.

Our data demonstrate that microglia are not the primary instigators but rather active participants contributing to rAAV-induced inflammation, responding to the presence of rAAV antigens and activity of recruited immune cells, and transitioning to an altered phenotype during the persistent phase. Considering potential alternative microglia-independent mechanisms that may initiate the response, several candidates within the tissue express TLRs 2/9, including Müller glia, retinal ganglion cells, and endothelial cells.^65,66^ Thus, future studies will inform our understanding of the extent to which rAAV transduction of different retinal cell constituents and the route of administration contribute to driving the innate response.^67^

The data also demonstrate the central role that chronic adaptive immune responses orchestrated by T cells directed against rAAV antigens play in GTAU. In humans, GTAU is typically seen within 1 month of IVT vector administration,^11,68,69^ particularly with higher vector doses, which is generally associated with a higher risk of post-operative inflammation. Taking an iterative approach to delineate the contribution and requirement of adaptive cells, we deployed RAG2 deficiency and S1PR modulation (FTY720 treatment) to unequivocally demonstrate that the recruitment of peripheral lymphocytes is both necessary and sufficient to drive the GTAU response following IVT rAAV administration. Animals lacking T and B cells do not exhibit clinical GTAU signs or changes in microglial phenotype (reduced or increased expression homeostatic and or activation markers) (Figure 5). Similarly, short-term pharmacological T cell blockade by FTY720 effectively prevented both cellular infiltrate and microglial activation, which, importantly, was maintained until 50 dpi following treatment withdrawal (Figures 6 and 7). Our finding that GTAU- and AAV-induced retinal microglial activation required peripheral T cell recruitment highlights the importance of recent work by Yin et al. implicating ocular draining lymphatics in immune recognition of IVT rAAV.^24^

Currently, prophylactic or reactive corticosteroid treatment regimens are employed for patients in subretinal and IVT rAAV-mediated retinal gene therapy trials to mitigate GTAU. Alternative corticosteroid-sparing treatments have been trialed sporadically for GTAU prophylaxis or recurrent/steroid-unresponsive inflammation, but no systemic approach to identify a safe and effective alternative to corticosteroids has been reported to date.^10,70^ We utilized a well-characterized mouse model of GTAU to screen a wide range of conventional and biologic immunomodulatory therapies for effective suppression of local and systemic markers of AAV-mediated immune activation. FTY720 (Gilenya), was identified as the most effective option in our screen. In support of our T cell-dependent model of GTAU disease pathogenesis, other T cell-directed therapies such as Tac and CsA also demonstrated a treatment effect, albeit less complete than that of FTY720. Surprisingly, despite pre-treatment for 1 month before AAV administration, neither MTX nor MMF were effective in preventing GTAU despite previously published benefit in EAU.^71^ Biologic therapies directed against TNF, IL-6, and B cells were not effective, suggesting that these cells and signaling molecules are redundant or not mechanistically important in GTAU. While not effective for preventing uveitis, B cell depletion did demonstrate a significant decrease in serum total anti-AAV binding antibodies. Further studies could explore the impact of B cell depletion in preventing the loss of transduction efficiency previously reported in fellow eyes after prior IVT injections and support flexible dosing strategies for bilateral treatments. The failure of the other agents tested here was not anticipated.^72^ All have demonstrated efficacy in preventing uveitis in EAU or other autoimmune disease models such as collagen-induced arthritis or experimental autoimmune encephalitis.^73–77^ This suggests that GTAU and other forms of EAU may be mechanistically distinct and may not respond to the standard stepladder immunosuppression approach used clinically to treat human uveitis.^78^ Of note, none of the agents were tested in combination with perioperative topical steroids, which would be the standard of care in most human clinical trials. Theoretically, this combination could have supported the efficacy of more agents.

One key finding of this study was the durable impact of short-term therapy with FTY720 on GTAU. FTY720 received breakthrough therapy designation as the first oral disease-modifying therapy approved for the treatment of patients with relapsing forms of multiple sclerosis (MS), including pediatric patients.^79,80^ In clinical use, FTY720 is well tolerated, with a favorable safety profile and efficacy in MS. Of particular concern to the ophthalmology community are reports from the two phase 3 registry studies for FTY720 that identified a dose-dependent incidence of macular edema (ME) in patients with MS.^81,82^ Importantly, for the lower (0.5 mg) dose that was ultimately approved for commercial use, ME occurred in less than 0.5% of patients, was usually asymptomatic, and resolved spontaneously upon cessation of treatment.^83–85^ Further exploration of this potential risk in the NHP model of GTAU (unpublished data) will help inform the decision to pursue treatment with perioperative FTY720 as a possible alternative to corticosteroids for GTAU prophylaxis in humans. Furthermore, the recent development of second-generation S1PR subtype-specific modulators has improved the safety profile of S1PR modulation and may provide alternatives that avoid the risk of ME.^86–88^ Future studies could also test novel compounds that modulate upstream and downstream members of the S1PR signaling pathway for confirmation of the role of the S1PR signaling in GTAU disease mechanisms and for efficacy in the absence of unwanted side effects.^89–101^

There are many inherent limitations in all comparative studies of gene therapy-associated inflammation. Key among these are the differences in immune function between species and the variability of gene therapy vectors used for research purposes.^102,103^ One strength of the present study is the demonstration that the mouse model of GTAU is robust and provided equivalent therapeutic efficacy data for Dex and FTY720 in two separate labs on two separate continents despite small variations in protocols and reagents. In the present study, the two labs used different AAV production facilities, different vector genomes (vg; null vs. GFP), final dose (vg/eye) and injection volume, method for detection of injection reflux, and mouse strains (C57BL6 vs. *Cx3cr1*^*CreER*^*:R26-tdTomato*^+/−^ reporter). However, despite these variations, the results were consistent in the control and treated conditions in both labs and support the rigor of the results. The relative contribution of recognized factors such as vector dose and route of administration that can influence GTAU risk and severity were not tested here; thus, it is not clear to what extent these results can be generalized to all situations.

In summary, our data provide new insight into both the early and late stages in the GTAU response to IVT rAAV. They further highlight the need for longitudinal studies to understand whether rAAV elicits a permanent alteration in ocular immune homeostasis that persists beyond the 50-dpi time point studied here.^58^ The next steps in understanding GTAU will need to extend to identify the full range of immune cell types involved in disease pathogenesis and to detail the reciprocal interactions between immune cells and the resident neuronal, glial, and stromal cells of the eye. Harnessing high-resolution multiplex imaging,^104,105^ immuno-phenotyping (extended flow cytometric panels), and single-cell transcriptomic approaches will permit a correlative and comprehensive evaluation of the distribution, phenotype (e.g., pathogenic or memory T cells), and relationship of the different immune cell populations remaining within the ocular compartments (anterior and posterior). These studies will continue to inform future approaches to enhance gene therapy-treatment efficacy and prevent unwanted tissue damage and sight-threatening sequelae such as GTAU.

## MATERIALS AND METHODS

### Study design

All experiments were designed and conducted in accordance with the Association for Research in Vision and Ophthalmology Statement for Use of Animals in Ophthalmic and Vision Research and relevant national legislation and policy. Specifically, this study com-plies with the Animals (Scientific Procedures) Act of 1986 under a UK Home Office Project License (PP9783504) and the US Public Health Service Policy on Humane Care and Use of Laboratory Animals, with approval from the University of Bristol Animal Welfare and Ethics Review Body and the Institutional Animal Care and Use Committee (IACUC) at the University of Washington (protocol no. 4481–02). Experiments included both male and female mice, labeled in all figures as circular and triangular points, respectively.

### Vector design, production, and handling

Vectors were kindly designed and plasmids provided by Ying Kai Chan (Harvard University, Cambridge, MA) or cloned in-house (Figures S10 and S11). Genomes were constructed on a single-stranded backbone flanked by inverted terminal repeats (ITRs), containing a cytomegalovirus (CMV) enhancer and promoter, an SV40 intron, a Woodchuck hepatitis virus posttranscriptional regulatory element (WPRE) sequence, a bovine growth hormone poly(A) signal, and, optionally, a TLR9-cloaking io2 sequence. Plasmids were amplified in One Shot TOP10 Chemically Competent *E. coli* (C404010; Thermo Fisher Scientific, Waltham, MA), extracted using a Monarch Plasmid Miniprep Kit (T1010L; New England Biolabs, Ipswich, MA) into 1× Tris-acetate-EDTA buffer (catalog no. 15558–026, Invitrogen, Waltham, MA), and quality checked by spectrophotometric analysis using a NanoDrop ND-1000 (Thermo Fisher Scientific) and whole-plasmid sequencing (Eurofins Genomics, Luxembourg City, Luxembourg).

Vectors were produced by VectorBuilder (Chicago, IL) at ultra-purified research grade by triple transfection of adherent HEK293 cells with Rep-Cap, E4/E2a/VA, and transfer plasmids, followed by polyethylene glycol (PEG) precipitation and CsCl gradient centrifugation. The quality of preparations was assessed by VectorBuilder, with viral titer assessed by qPCR targeted at the ITRs, sterility determined via inoculation into culture medium, mycoplasma contamination evaluated via inoculation onto indicator cells followed by FS microscopy, viral purity determined at >80% by SDS-PAGE, endotoxin contamination evaluated by limulus amebocyte lysate (LAL) testing at <10 endotoxin units (EU)/mL, with a typical full capsid ratio assessed by electron microscopy as >70%. All preparations were stored in 200 mM NaCl or PBS (catalog no. D8537, Sigma-Aldrich, Burlington, MA), with 0.001% Pluronic F-68 (catalog no. 24040–032, Thermo Fisher Scientific) at −80°C, and two freeze-thaw cycles before use for handling purposes.

Alternatively, vectors were produced by the Gene Therapy Center Vector Core at the University of Massachusetts Chan Medical School. Briefly, production was performed by triple transfection of HEK293 cells, followed by purification via PEG precipitation and CsCl gradient centrifugation. For quality control, the following parameters were assessed by the facility: viral titer tested by ddPCR targeted at the CMV promoter, mycoplasma contamination evaluated via PCR, viral protein ratios and vector purity determined by SDS-PAGE, and endotoxin contamination evaluated by LAL testing at <0.5 EU/mL. Preparations were stored in PBS with 5% D-sorbitol and 0.001% Pluronic F-68 at −80°C, with no more than two freeze-thaw cycles before use for handling purposes. For a convenient comparison of differences in vector production, see Table S28.

### Animals

Microglial reporter C57BL/6J *Cx3cr1*^CreER^*:R26-tdTomato*^+/−^ and lymphocyte-deficient C57BL/6J *Rag2*^−/−^ (B6.Cg-*Rag2*^*tm1.1Cgn*^/J) mice were supplied from established breeding colonies at the University of Bristol, and WT C57BL/6J mice were purchased from Charles River Laboratories (UK and US locations).^31^ Homozygous *Cx3cr1*^*CreER*^*:R26-tdTomato* mice were mated with C57BL/6J mice to generate heterozygous reporter mice for experiments. To activate tdTomato expression in the retinal microglia, animals received eye drops containing 5 mg/mL tamoxifen (catalog no. T5648, Sigma-Aldrich) three times per day (every 2–3 h) for 3 consecutive days, followed by a minimum 3-week washout period before using animals in experiments. This allowed for increased specificity of microglial labeling.^36^ All mice were used at 6–12 weeks of age. All mouse strains were confirmed as negative for the *Rd8* mutation and were housed under specific pathogen-free conditions with food and water *ad libitum*.^106^

### IVT injection

Mice were anesthetized by inhalation of 1.5%–2.0% nebulized isoflurane (catalog no. 988–3245, Henry Schein, Melville, NY). Pupils were dilated by topical administration of tropicamide 1% w/v (catalog no. PL 03468/0084, Bausch + Lomb, Kingston-Upon-Thames, UK) and phenylephrine hydrochloride 2.5% w/v (catalog no. PL 03468/0076; Bausch + Lomb), and ocular hydration was maintained with Viscotears Liquid Gel (catalog no. PL 13757/0020, Bausch + Lomb). We delivered 2 μL viral suspension at 5E12 gc/mL for a dose of 1E10 gc/eye or vehicle (PBS with 0.001% Pluronic F-68 v/v [catalog no. 24040–032, Gibco, Waltham, MA]) into the IVT space via the pars plana, using an operating microscope and a 33G needle on a microsyringe (Hamilton Company, Reno, NV) under direct visualization. In brief, each globe was proptosed and immobilized using forceps to aid in the insertion of the needle, approximately 2 mm circumferential to the corneal limbus at a 45° injection angle. When the needle bevel became visible within the vitreous, the forceps were released, and 2 μL viral suspension was injected slowly by a human operator. Following injection, the needle was held in position (15 s), allowing the transient increase in intraocular pressure to visibly equalize before retraction. Direct visualization of the procedure allowed the needle operator to monitor for signs of fluid reflux from the injection site, which served as an exclusion criterion from analysis. Immediately following injection, 1% w/w chloramphenicol ointment (catalog no. PL00156/0363, Martindale Pharmaceuticals, Romford, UK) was applied topically to the eye, with the animals monitored and placed on a heating pad at 41°C for recovery.

Alternatively, for experiments (Figures 6A, 6B, 7E–7G, and S6A–S6E), anesthesia was induced by intraperitoneal (i.p.) injection of 6.9 mg/kg body weight of a 1% solution comprising 100 mg/mL ketamine (NDC 54771-2013-1, Zoetis, Parsippany, NJ) and 20 mg/mL xylazine (catalog no. NADA 139–236; Akorn, Lake Forest, IL) in sterile water. Local anesthesia was induced by topical administration of 0.5% v/v tetra-caine hydrochloride (NDC 68682-920-64, Oceanside Pharmaceuticals, Bridgewater, NJ), infection prevented by 5% v/v topical betadine (NDC 0065-0411-30, Alcon Laboratories, Fort Worth, TX), dilation achieved by 2.5% v/v topical phenylephrine hydrochloride (NDC 70756-649-35, Lifestar Pharma, Mahwah, NH), and hydration maintained by Genteal Tears lubricant eye gel (catalog no. 25368103, Alcon Laboratories). The animal was placed on a custom-made stereotaxic apparatus, and a guide hole in the sclera, 1–2 mm posterior to the limbus, was created using a sterile 30G needle. To induce GTAU, 1.2 μL of a solution comprising 2.7E12 gc/mL AAV2.CMV.eGFP:WPRE in PBS (catalog no. 14200075; Thermo Fisher Scientific) with 0.03% w/v clinical-grade fluorescein sodium (NDC 17478-253-10, AK-FLUOR, Akorn, Lake Forest, IL) tracer dye for a dose of 5.4E9 gc/eye, or vehicle, were delivered into the posterior chamber by a 34G needle (catalog no. NF34BV, World Precision Instruments, Sarasota, FL) attached to a microsyringe (NANOFIL, World Precision Instruments) and a micropump (catalog no. 062403, World Precision Instruments). To control for and mitigate potential reflux, vector was delivered at 40 nL/s within four cycles of 400 nL, interspaced with pauses to allow intraocular pressure to decrease, with visible reflux of fluorescein dye serving as an exclusion criterion from analysis. Drinking water was supplemented with 200–300 mg/kg acetaminophen (Children’s Tylenol Oral Suspension, Kenvue, Wilmington, DE) for 30 days following injection to minimize discomfort, as set by the IACUC at the University of Washington. For a convenient comparison of techniques employed, see Table S29.

### Intraocular imaging and inflammation severity measurement

Ocular dynamics were investigated by *in vivo* microscopy of the retinal fundus in bright-field and FS configurations and OCT of the retina via the Micron IV imaging system (Phoenix-Micron, Bend, OR) under pupil dilation and isoflurane anesthesia. Images were acquired at standardized settings, with bright-field images captured at 15 frames per second (fps) and +3 dB gain and FS images captured at 2 fps and gain of −2 to +18 dB. Image analysis of tdTomato mean FS intensity (MFI) and OCT circle scan retinal thickness was performed using the “Measure” function and InteredgeDistance version 2.0 macro within Fiji,^107,108^ respectively. To quantify retinal thickness, the perpendicular distance between the ganglion cell layer and retinal pigment epithelium was assessed at 25 equidistant locations spanning the longitudinal extent of the tissue scan (Figure S12).

Alternatively, for experiments in Figures 7E and 7F, OCT scans (1,000 A-/B-scans, 20× averaging) of the anterior chamber and retina were captured under pupil dilation and ketamine/xylazine anesthesia using the Bioptigen Envisu R2300 (Bioptigen, Morrisville, NC). For the anterior chamber, a 12-mm telecentric lens (catalog no. 90-BORE-G3–12, Bioptigen) was used to capture a 3.6-mm vertical B-scan centered on the pupil. For the retina and vitreous, a mouse retina lens (catalog no. 90-BORE-G3-M, Bioptigen) was used to capture a 1.6-mm B-scan centered on the optic nerve.

Quantification of cellular infiltration was performed by an investigator blinded to all treatment conditions, capturing the number of bright opaque objects present in the vitreous chamber on an OCT B-scan by use of included ImageJ functions, with a method informed by the previously published protocol by Chu et al.^109^ Specifically, a region of interest within the vitreous between the inner limiting membrane and the lens was defined using the “Polygon Selection” tool, and opacities were counted using the “Find Maxima” feature, allowing the use of a prominence (previously noise tolerance) in the range of 13–18 (Figures 1B, 4B, 5B, and 6D) or 13–15 (Figure 7F) to ensure counting accuracy in the presence of speckle noise (Figure S13). To mitigate greater noise in OCT scans captured via the Micron IV system from animals under isoflurane anesthesia (Figures 1B, 4B, 5B, and 6D), image encoding was converted from 16- to 8-bit depth and smoothed before quantification via the “Image > Type” and “Process > Smooth” functions in ImageJ, respectively. All machine quantifications were confirmed by a human investigator to ensure reliability.

### Confocal FS microscopy of retinal flatmounts

Enucleated eyes were incubated in 4% w/v paraformaldehyde (PFA; catalog no. 28908; Thermo Fisher Scientific) for 30 min at 4°C, dissected into eye cups, and further incubated in a 1:4 dilution of BD Cytofix/Cytoperm (catalog no. 554722; BD Biosciences, Franklin Lakes, NJ) in PBS overnight at 4°C. Following a wash in PBS, the retinae were removed from their eye cups and incubated for 24 h on a shaker at room temperature (RT) in a 48-well plate containing 1× BD Perm/Wash (catalog no. 554723, BD Biosciences), 5% normal donkey serum (catalog no. ab7475, Abcam, Cambridge, UK), and 1:100 anti-CD16/32 F_C_ block (catalog no. 553142, BD Biosciences) at 300 μL per well. Subsequently, the retinae were stained with primary antibodies diluted in 300 μL of 1× BD Perm/Wash for 1–3 days at RT on a shaker, minimizing light exposure where possible. Following three washes in PBS over 3 h at RT, the retinae were stained with secondary antibodies diluted in 300 μL of 1× BD Perm/Wash for 24 h at RT, if necessary, and subsequently incubated in 1% w/v PFA for 15 min at 4°C. Each retina was mounted, under a dissection microscope, in the center of an imaging spacer (catalog no. T-03, GVB-geliMED, Bad Segeberg, Germany) on a microscope slide, flattened via three radial incisions and trimming of excess peripheral tissue, immersed in Ce3D Tissue Clearing Solution (catalog no. 427703, BioLegend, San Diego, CA), protected by a cover slide (catalog no. 631–0125, VWR International, Radnor, PA), sealed with nail polish, and incubated overnight at RT for successful tissue clearing. Samples were imaged within 3 days of mounting on a Leica SP5-II AOBS confocal laser scanning microscope attached to a Leica DMI6000 inverted epifluorescence microscope (Leica Microsystems, Wetzlar, Germany).

### Treatment interventions

Microglial depletion: PLX5622 free base (CAS 1303420-67-8; catalog no. C-1521, Chemgood, Henrico, VA) was integrated into the AIN-76A rodent diet (catalog no. D10001i, Research Diets, New Brunswick, NJ) at 1,200 mg/kg (catalog no. D19101002i, Research Diets).^25^ Mice were provided *ad libitum* access to PLX5622 or control diets throughout.

Pharmacological treatments: The specific dosing regimens (drug concentration, frequency, duration, and route of administration) were based on previous publications as referenced.

FTY720 hydrochloride (6 mg/kg) (CAS 162359-56-0; catalog no. 10006292, Cayman Chemical, Ann Arbor, MI) or vehicle (droplet digital H_2_O with 0.167% v/v DMSO; catalog no. 276855, Sigma-Aldrich) was orally dosed to mice every other day using a flexible plastic feeding tube (catalog no. FTP-20-38, Instech, Plymouth Meeting, PA) attached to a 1-mL syringe (catalog no. MDSS01SE, Terumo, Tokyo, Japan).^56,57^ Alternatively, for experiments (Figures 6A, 7E–7G, and S6A–S6F), FTY720 hydrochloride in sterile water (3 mg/kg) (Sigma SML0700) was administered i.p. daily, from 0 to 28 dpi.^110^

Dex sodium phosphate (CAS 2392-39-4; NDC 67457-420-00; Mylan, Morgantown, WV) formulated in PBS was delivered i.p. every day according to the following tapered regimen: 2 mg/kg from −2 to 7 dpi, 1 mg/kg from 8 to 14 dpi, 0.5 mg/kg from 15 to 21 dpi, and 0.25 mg/kg from 22 to 28 dpi. CsA (20 mg/kg) (CAS 59865-13-3; catalog no. HY-B0579, MedChemExpress, Monmouth Junction, NJ) and Tac (3 mg/kg) (CAS 104987-11-3; catalog no. HY-13756A, MedChemExpress) formulated in PBS with 5% DMSO and 0.1% Tween 20 (catalog no. BP337–100, Thermo Fisher Scientific) were both administered i.p. every day from −7 to 28 dpi.^111,112^ Pio (5.3 mg/kg) (CAS 111025-46-8, catalog no. B1117, APExBIO Technology, Houston, TX), and Fen (20 mg/kg) (CAS 49562-28-9; catalog no. F6020, Merck, Darmstadt, Germany) formulated in 5% DMSO, 40% PEG300 (catalog no. 90878, Sigma-Aldrich), 5% Tween-80 (CAS 9005-65-6, catalog no. 11441981, Fisher Scientific), and 50% saline (0.9% NaCl w/v in water) were administered i.p. from −1 to 28 dpi.^51,52^ Control groups received corresponding vehicle formulation.

MTX (CAS 59-05-2; NDC 0703-3671-01; Teva Pharmaceuticals, Parsippany, NJ) and MMF (CAS 116680-01-4; NDC 17478-422-40; Akorn) were delivered *ad libitum* supplemented in drinking water at 6.25 μg/mL and 0.625 mg/mL from −28 to 28 dpi as previously described.^71^ For monoclonal antibodies: anti-mouse CD20 (20 mg/kg) (clone MB20–11; catalog no. BE0356, BioXCell, Lebanon, NH) was administered i.p. weekly from −7 to 28 dpi^113^; anti-mouse TNF-α (10 mg/kg) (clone XT3.11; catalog no. BE0058, BioXCell) was administered i.p. from −1, 0, and 1 dpi and then alternate days until 28 dpi^114^; mouse anti-dengue virus IgG2c (immunoglobulin G2c) isotype control (catalog no. BE0366, BioXCell) formulated in InVivoPure pH 7.0 Dilution Buffer (catalog no. IP0070, BioXCell) for anti-CD20 or anti-TNF-α administered following each regimen; and anti-mouse IL-6 (1 mg/kg) (clone MP5–20F3; catalog no. 16-7061-81; Thermo Fisher Scientific) formulated in PBS was administered i.p. on −1, 1, 3, and 7 dpi.^115^

### Flow cytometry

According to our established method, retinae and vitreous humor were harvested by ocular dissection in 100 μL ice-cold PBS (catalog no. D8537, Sigma-Aldrich) via limbal incision, lens and anterior chamber removal, and transfer into a 1.5-mL microcentrifuge tube.^5,36^ Subsequently, samples were processed into single-cell suspensions by mechanical disruption and filtered through a 60-μm nylon mesh filter plate (catalog no. MANMN6010, Sigma-Aldrich) centrifuged at 400 × *g* for 5 min, with the supernatant aspirated and cell pellet resuspended in Cell Staining Buffer (catalog no. 420201; BioLegend) prior to F_C_ receptor inhibition (20 min at 4°C) and staining with fluorophore-conjugated monoclonal antibodies and viability dye (30 min at 4°C) (Table S30). For tdTomato reporter compensation, cell suspensions of *Cx3cr1*^CreER^:*R26*-*tdTomato*^+/−^ heterozygote brains were used. FS-minus-one (FMO) controls of cerebral tissue were utilized for gating of microglial activation markers. Samples were acquired on a five-laser BD LSRFortessa X-20 Cell Analyzer (BD Biosciences) and analyzed using FlowJo version 10.8.1 (BD Biosciences) (Figures S5 and S6). Seven 2-fold serial dilutions of a known concentration of splenocytes were acquired at identical settings as retinal samples to construct a standard curve and calculate absolute cell numbers.

Alternatively, for experiments (Figures 6A, 6B, 7G, and S6A–S6C), individual eyes were dissected in 50 μL ice-cold fluorescence-activated cell sorting (FACS) buffer (PBS with 2% v/v FBS) to collect intraocular contents (retina, choroid, aqueous, vitreous, and uveal tissue) after the removal of lens, sclera, and cornea. Each sample was placed in 100 μL FACS buffer, manually disrupted with Vannas scissors (catalog no. S7–1375; Stephens Instruments, Lexington, KY), transferred to a 1.5-mL microcentrifuge tube, and incubated with 10 mg/mL DNAse (catalog no. 11284932001, Roche Diagnostics, Mannheim, Germany) and 0.5 mg/mL collagenase D (catalog no. 11088858001, Roche Diagnostics) at 37°C for 25 min. A single-cell suspension was generated by filtration through a 70-μm mesh filter plate, washed with FACS buffer, and transferred to a 96-well V-bottom plate. For staining, 1E6 cells per sample were incubated in 2% TruStain FcX anti-mouse CD16/32 antibody (catalog no. 101320; BioLegend) for 15 min at 4°C, followed by incubation with fluorophore-conjugated monoclonal antibodies (Table S31) for 25 min at 4°C. After two washes in FACS buffer, samples were incubated with 7-aminoactinomycin D for 10 min at 4°C. Compensation was performed using single-color controls prepared with UltraComp eBeads (catalog no. 01-2222-42, Thermo Fisher Scientific) or splenocytes. Cell counts were determined using CountBright absolute counting beads (catalog no. C36950, Thermo Fisher Scientific) according to the manufacturer’s protocol. FMO controls were used to assist in the gating of the markers selected for validation. Un-stained cell controls were used to determine marker-negative cell populations. Samples were acquired on a BD FACSymphony A3 Cell Analyzer (BD Biosciences) and analyzed using FlowJo version 10.1 (BD Biosciences).

### Bioinformatics

Raw data of microglial transcriptomes from 3-month-old *Cx3cr1*^*CreER*^*: R26-tdTomato*^+/−^ mice IVT injected with AAV2.CMV.Null.WPRE.io2 (1E10 gc/eye) were obtained from the Gene Expression Omnibus (GEO) repository (GSE266332).^20^ Sequence data were concatenated using the “cat” Unix utility and quality checked with FastQC,^116^ followed by paired-end adapter removal (Nextera XT Index Kit version 2 adapter sequences as reference) and base trimming from the 3′ end, initially by a single base to a 75-base total length and further by a 4-base sliding window approach based on a minimum quality (Phred) score of 30 using Trimmomatic,^117^ and concluded by final post-trim FastQC analysis. Alignment and counts generation were performed by Salmon 1.10.1 in mapping-based selective alignment mode with sequence-specific and fragment-level GC bias correction,^118^ against a full decoy Salmon index based on the Ensembl Release 108 cDNA (excluding non-coding RNA) sequence and the GRCm39 DNA primary assembly. Further diagnostic information was generated using RNA STAR with featureCounts,^119,120^ utilizing Ensembl Release 108 gene annotations and the GRCm39 DNA primary assembly as reference, followed by a quality check of all results using QualiMap 2 and MultiQC (Figure S3).^121,122^ Salmon outputs were analyzed for differential gene expression by DESeq2, following gene-level import by tximeta,^123,124^ with a gene considered DEG provided a false discovery rate (FDR) step-up *q* value of ≤0.05 and a fold change value of ≥1.5. Batch correction was performed using dds design parameters in DESeq2 for DEG analysis and using ComBat-seq for transcripts per kilobase million-normalized Salmon counts.^125^

Functional analysis for enrichment of Gene Ontology (GO) terms and Kyoto Encyclopedia of Genes and Genomes pathways was performed using WebGestalt 2024 in gene set enrichment analysis mode and ShinyGO 0.80.0 utilizing overrepresentation analysis,^126,127^ with non-redundant terms or pathways considered enriched provided an FDR step-up *q* value of ≤0.05. Additional functional insights were gained using Ingenuity Pathway Analysis (QIAGEN, Hilden, Germany), content version 122103623 and building_quartz, employing the “ingenuity knowledge base (genes only)” reference set, both interaction and causal networks, “all” node types, “all” data sources, “experimentally observed” settings for microRNA confidence, “all” species, “all” tissues and cell lines, and “all” mutations, with pathways considered enriched provided a *p* value of 0.1.

### Detection of total anti-AAV capsid binding antibodies in serum

Blood was collected by cardiac puncture postmortem and centrifuged to collect serum. Indirect ELISA assays were performed to detect antibodies against AAV capsid in serum, as described previously.^128,129^ Briefly, a 96-well flat-bottom plate (catalog no. 44-2404-21, Thermo Fisher Scientific) was coated with empty AAV2 capsid (catalog no. 66V020, Progen, Heidelberg, Germany) at 5E9 capsids per well and incubated overnight at 4°C. Following three washes in PBS with 0.05% v/v Tween 20 (catalog no. BP337–100, Thermo Fisher Scientific), the plate was blocked with 200 μL PBS with 5% BSA (catalog no. 12659-M, Sigma-Aldrich) for 2 h and then incubated with mouse serum (1:100, 100 μL/well) for 1 h at 20°C. After a further round of washing, the plate was incubated with 1:20,000 horseradish peroxidase-conjugated goat anti-mouse IgG + IgM + IgA H&L antibody (catalog no. ab102448, Abcam) at 100 μL/well for 1 h at 20°C.

Subsequently, the plate was incubated with TMB ELISA Substrate (High Sensitivity) (ab171523, Abcam) for 10 min, and the reaction was halted with ELISA Stop Solution (catalog no. SS03, Thermo Fisher Scientific). Lastly, absorbance was measured on a plate reader (BioTek Synergy 2, BioTek Instruments, Winooski, VA) at 450 nm. Anti-AAV2 mouse monoclonal antibody (catalog no. 610298, Progen) was used to establish positive controls and a standard curve. All values were determined in duplicate.

### Statistical analysis

Data were compiled and analyzed using FlowJo 10.8.1, Microsoft Excel, GraphPad Prism 10.3.1, R 4.4.2, and ARTool 0.11.1.^130,131^ All datasets were assessed for normality using the Shapiro-Wilk test and visual examination of Q-Q plots. Transformation by x = log_10_(y) was trialed for all non-parametric data, followed by a further normality assessment. Where employed, results of log-transformed data analysis are displayed on visualizations of pre-transformation (linear) data. For comparisons with one independent variable (Figures 1, 2, 3, and S7A–S7F), parametric and non-parametric data were analyzed by a Brown-Forsythe and Welch ANOVA or Kruskal-Wallis test, respectively. In 2 × 2 factorial designs (Figures 4, 5, 6, 7, S2, S7G, and S7H), parametric and non-parametric data were analyzed by a two-way ANOVA or a factorial aligned rank transform (ART) ANOVA, respectively.^131^ Multiple comparisons testing was performed by FDR control with a *Q* threshold of 0.05, using the two-stage linear step-up procedure of Benjamini, Krieger, and Yekutieli.^132,133^ Where unavailable due to limitations of the ARTool package, the original method of Benjamini and Hochberg was utilized.^134^ In Figure S8, parametric and non-parametric data were analyzed by a Welch *t* test or a Mann-Whitney test, respectively. Effect size was reported as mean difference ± standard deviation. Samples with surgical trauma or lack of model response were excluded from analysis.

## Supplementary Material

Supplemental figures

Supplemental tables

Supplemental information can be found online at https://doi.org/10.1016/j.ymthe.2026.02.018.

## Data Availability

The mRNA-seq dataset analyzed in this study can be found at the GEO repository, accession number GSE266332. All other raw data will be made available by the authors at request.
