# Supplementary material for: Microglia-independent rAAV-induced inflammation causes persistent ocular immune dysregulation rescued by S1P receptor modulation
Source: Mol Ther. Author manuscript; Available in PMC 2026 Jun 17. (PMC13154287; doi:10.1016/j.ymthe.2026.02.018)
Supplement: Supplemental figures [file NIHMS2178743-supplement-Supplemental_figures.pdf]

YMTHE, Volume *34*

Supplemental Information

Microglia-independent rAAV-induced inflammation causes persistent ocular immune dysregulation rescued by S1P receptor modulation

Philip M. Langer, Alison J. Clare, Xudong Peng, Katherine L. Costello, Suci Cendanawati, Leslie L. Wilson, Amy Ward, Oliver H. Bell, Colin J. Chu, Ying Kai Chan, Andrew D. Dick, Kathryn L. Pepple, and David A. Copland

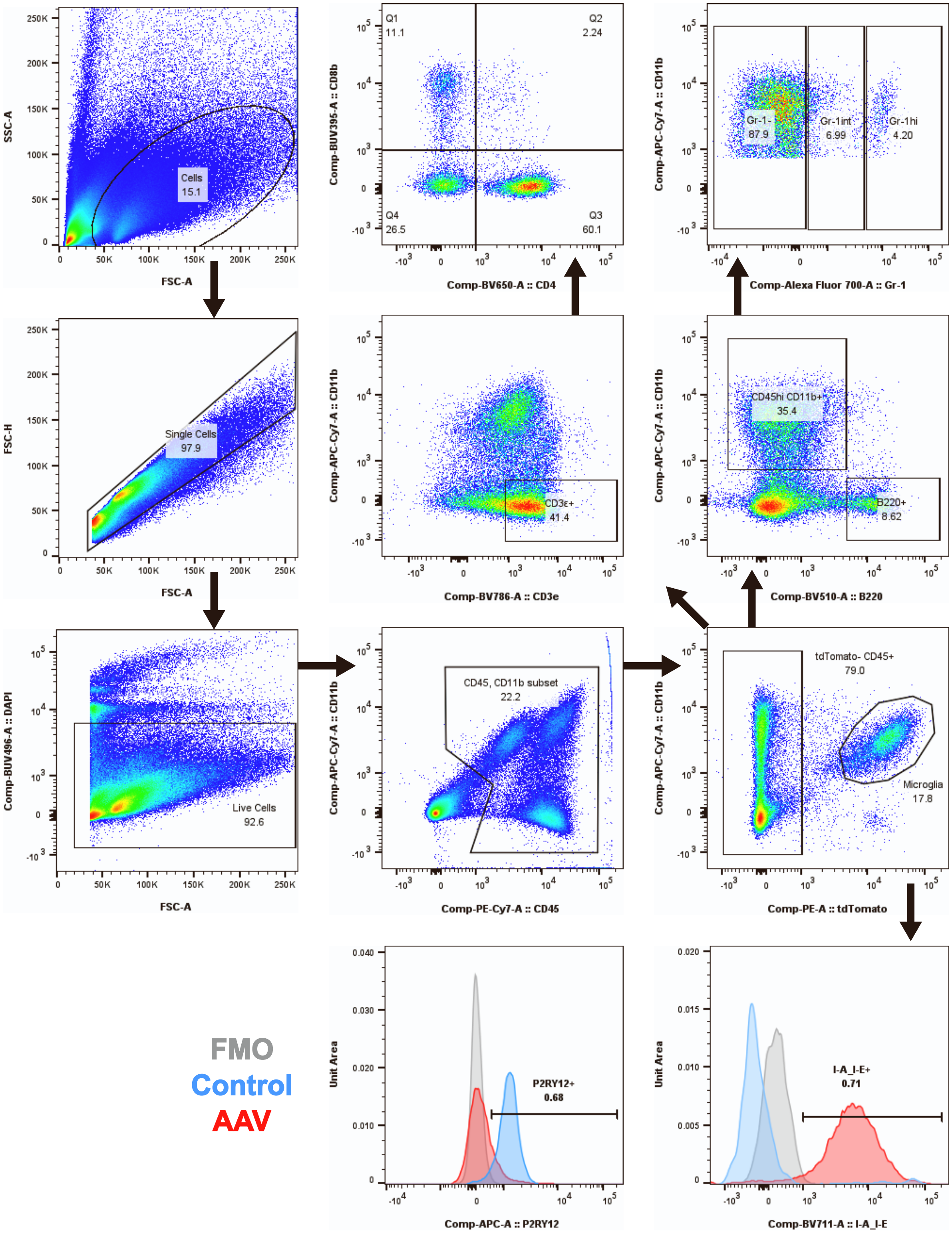

Figure S1. ***Cx3cr1^{CreER}:R26-tdTomato$^{+/-}$* mouse flow cytometry gating strategy.**

Cx3cr1^{CreER}:R26-tdTomato$^{+/-}$ C57BL/6J mice were intravitreally injected with AAV2.CMV.null:WPRE.io2 (1E10 gc/eye) at 0 days post-injection (dpi). Retina and vitreous from single eyes were processed into single-cell suspensions and stained with viability dye and fluorescent monoclonal antibodies for flow cytometric analysis at 12 dpi. Samples were gated for cells, singlets, live cells, and immune cell subtypes. Expression of homeostasis and activation markers on microglia (CD45^{int} CD11b^{+} tdTomato^{+}) was gated based on fluorescence-minus-one (FMO) controls reported as rate of expression (% microglia). Fluorescence channel spillover was corrected by compensation in BD FACSDiva using single-stain controls. Data was acquired on a BD LSRFortessa X-20 Cell Analyzer and analyzed in FlowJo 10.8.1.

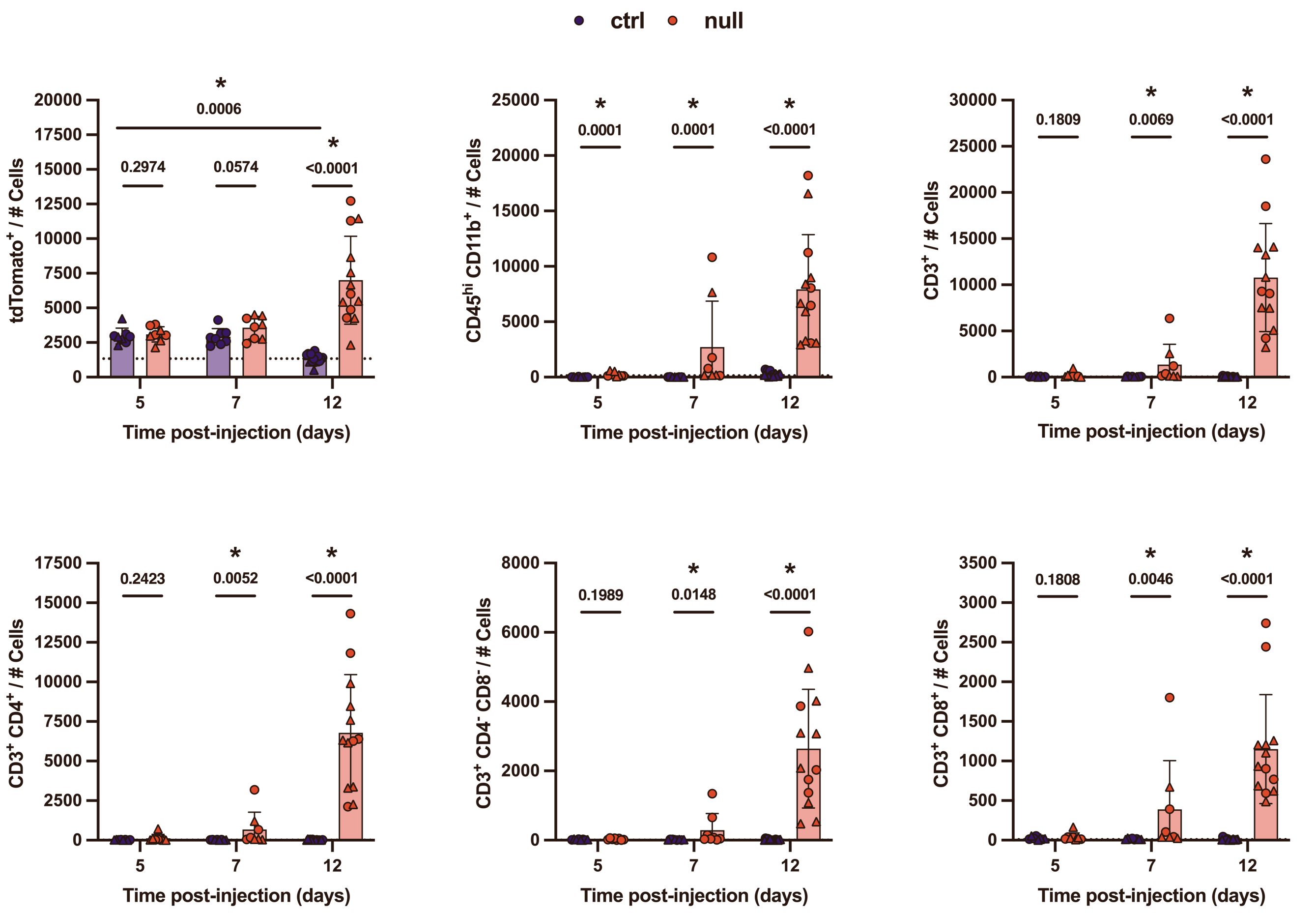

Figure S2. Injection trauma and vector-based effects on immune cell populations during acute period of rAAV-induced GTAU.

Cx3cr1^{CreER}*:R26-tdTomato*$^{+/-}$ C57BL/6J mice were intravitreally injected with AAV2.CMV.null:WPRE.io2 (null; 1E10 gc/eye) or control (ctrl; PBS with 0.001% v/v Pluronic F-68) at 0 days post-injection (dpi). Retina and vitreous from single eyes were processed into single-cell suspensions for multicolor flow cytometric analysis at selected time points. Absolute counts of resident tdTomato^{+} cells and infiltrating CD45^{hi} cell populations at 5 (n = 6-8), 7 (n = 6-8), and 12 (n = 11-15) dpi during rAAV-induced GTAU. Dashed line indicates the mean baseline number of tdTomato^{+} microglia found in naïve eye. Statistical analysis was performed by multiple Mann-Whitney tests with False Discovery Rate (FDR; Q = 0.05) correction using the two-stage step-up method of Benjamini, Krieger, and Yekutieli. * indicates q ≤ 0.05. Columns and error bars represent mean ± SD, respectively.

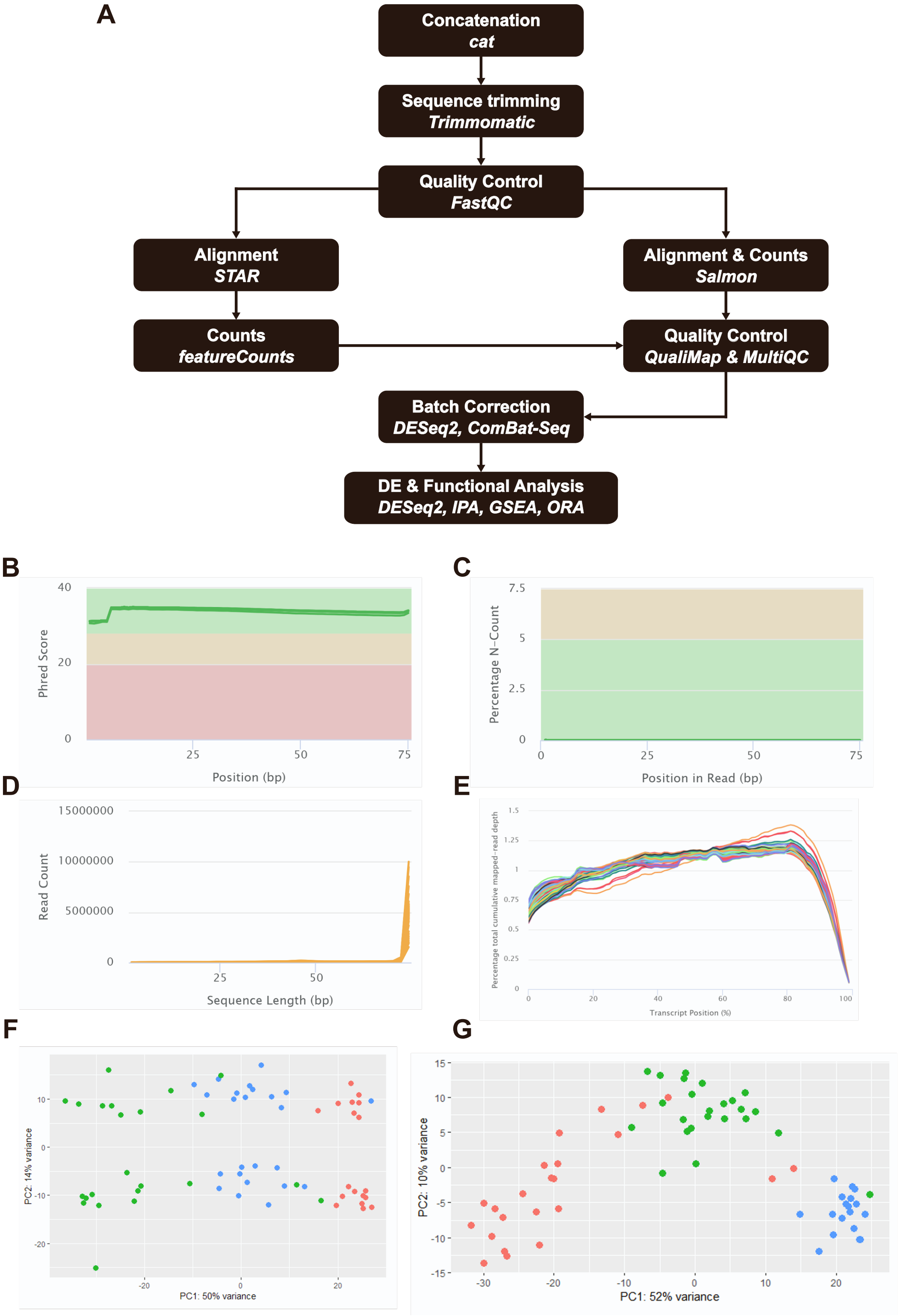

Figure S3. Workflow of microglia mRNA-Seq re-analysis.

Raw data of microglial transcriptomes from 3-month-old *Cx3cr1^{CreER}:R26-tdTomato$^{+/-}$* mice intravitreally injected with AAV2.CMV.eGFP:WPRE (1E10 gc/eye) at 0 days post-injection (dpi) and collected from naïve, 12, and 29 dpi was obtained from the Gene Expression Omnibus repository (GSE266332).[20] **(A)** Analysis workflow diagram, illustrating key steps and associated R packages. **(B)** Mean Phred score across each base along the length of all 75-base-long reads, indicating the quality of base calling during sequencing. **(C)** Percentage of base calls at each position for which an N (unknown base) was called. **(D)** Distribution of fragment sizes (read lengths) found in each sample, with a target of 75. **(E)** Transcript coverage profile, illustrating sequence 3' or 5' bias, with a ratio of 1.07 ± 0.05 in the presented dataset. **(F)** Principal component analysis (PCA) plot of samples collected in two batches, separated by time in PC1 and batch in PC2. **(G)** PCA plot of samples from Panel F following batch correction using ComBat-seq. Statistical analysis of differentially expressed genes (DEGs) was performed by DESeq2, with a significance threshold of fold change ≥ ± 1.5 and a Benjamini-Hochberg False Discovery Rate (FDR) q value of ≤ 0.05. Panels B-D generated by FastQC and visualized in MultiQC. Panel E generated by QualiMap and visualized in MultiQC. Panel G generated by DESeq2 and visualized using ggplot2.

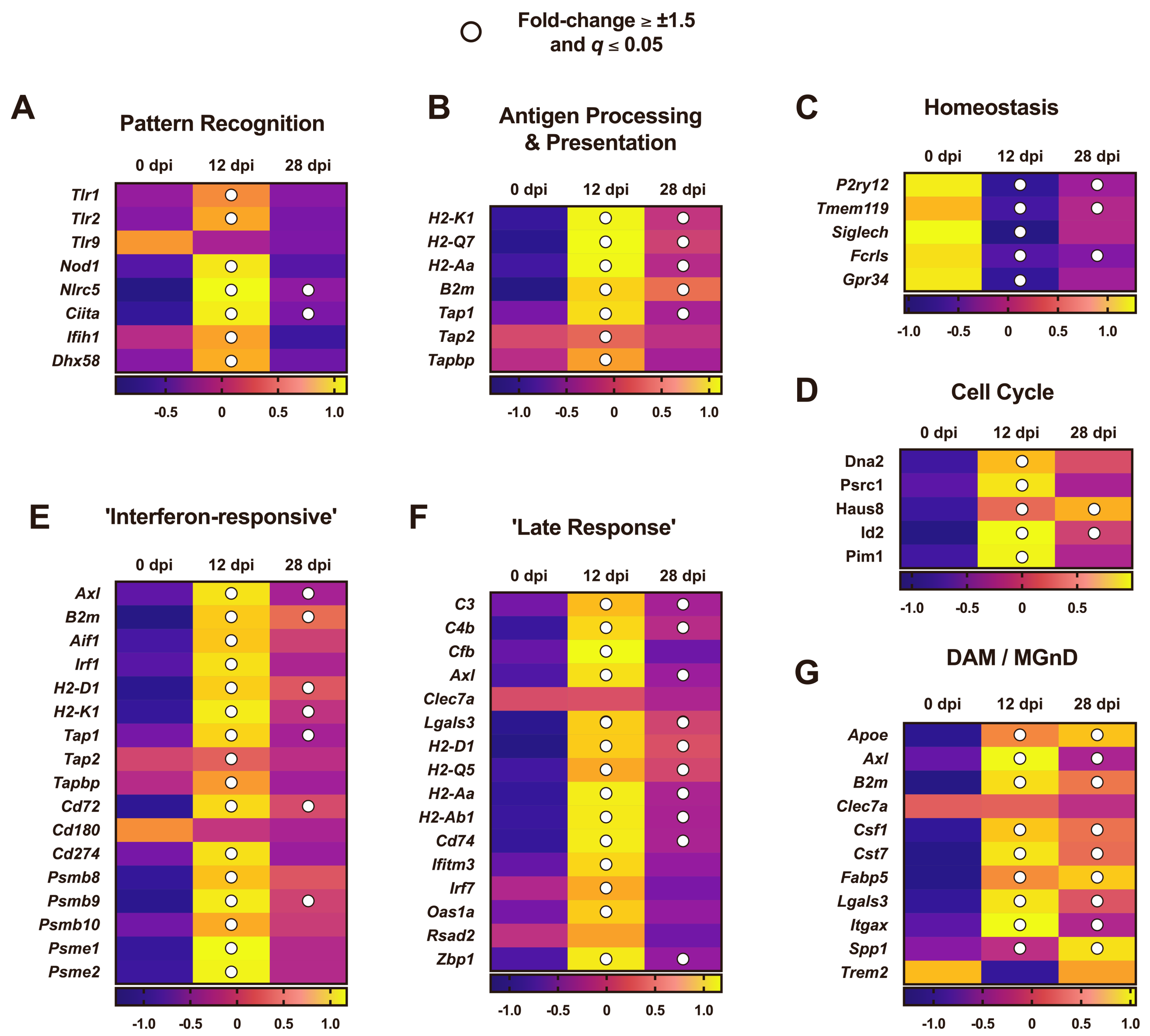

Figure S4. Transcription of additional microglial functional modules.

Raw data of microglial transcriptomes from 3-month-old *Cx3cr1*^{CreER}*:R26-tdTomato*$^{+/-}$ mice intravitreally injected with AAV2.CMV.eGFP:WPRE (1E10 gc/eye) at 0 days post-injection (dpi) and collected at 0, 12, and 29 dpi was obtained from the Gene Expression Omnibus repository (GSE266332), processed to generate normalized counts and identify differentially expressed genes (DEGs).[20] Genes were grouped based on category (e.g. pattern recognition) or membership in a previously published 'functional module' associated with a disease state (e.g. disease-associated microglia; DAM). Statistical analysis of DEGs was performed by DESeq2, with a significance threshold of fold change ≥ ± 1.5 and a Benjamini-Hochberg False Discovery Rate (FDR) q value of ≤ 0.05. Processed read values in transcripts per million (TPM) were generated by Salmon and z-scores computed to indicate deviation from the mean, illustrated as heatmap colors.

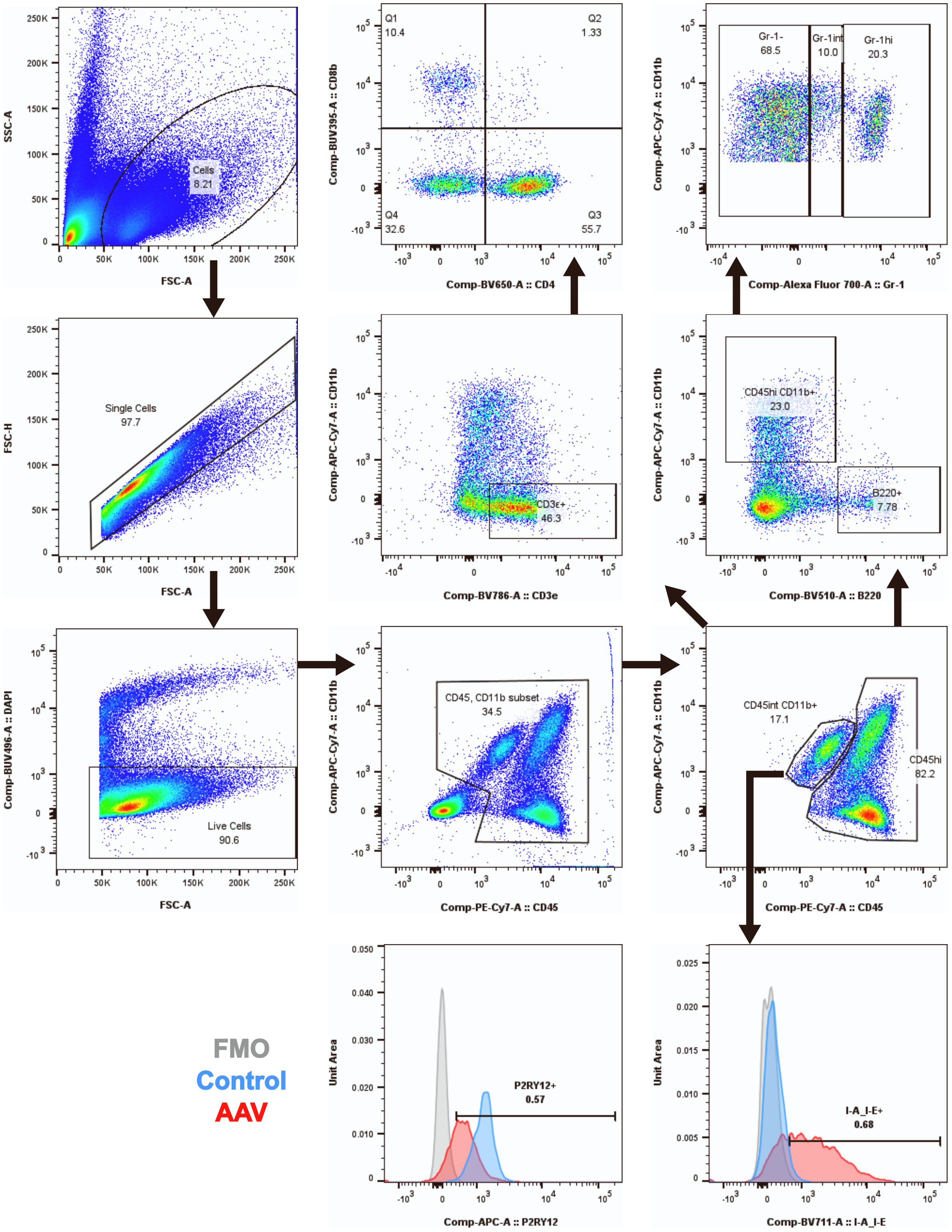

Figure S5. C57BL/6J wild-type and B6.Cg-Thy1 Rag2 knock-out mouse flow cytometry gating strategy. C57BL/6J (wild-type) and B6.Cg-Thy1 (Rag2 knock-out) mice were intravitreally injected with AAV2.CMV.null:WPRE.io2 (1E10 gc/eye) at 0 days post-injection (dpi). Retina and vitreous from single eyes were processed into single-cell suspensions and stained with viability dye and fluorescent monoclonal antibodies for flow cytometric analysis at 12 dpi. Samples were gated for cells, singlets, live cells, and immune cell subtypes. As these strains lack the *Cx3cr1^{CreER}:R26-tdTomato* allele, microglia were approximately phenotyped based on CD45^{int} CD11b^{+} for flow cytometry. Expression of homeostasis and activation markers on microglia was gated based on fluorescence-minus-one (FMO) controls and reported as rate of expression (% microglia). Fluorescence channel spillover was corrected by compensation in BD FACSDiva using single-stain controls. Data was acquired on a BD Fortessa X-20 Cell Analyzer and analyzed in FlowJo 10.8.1.

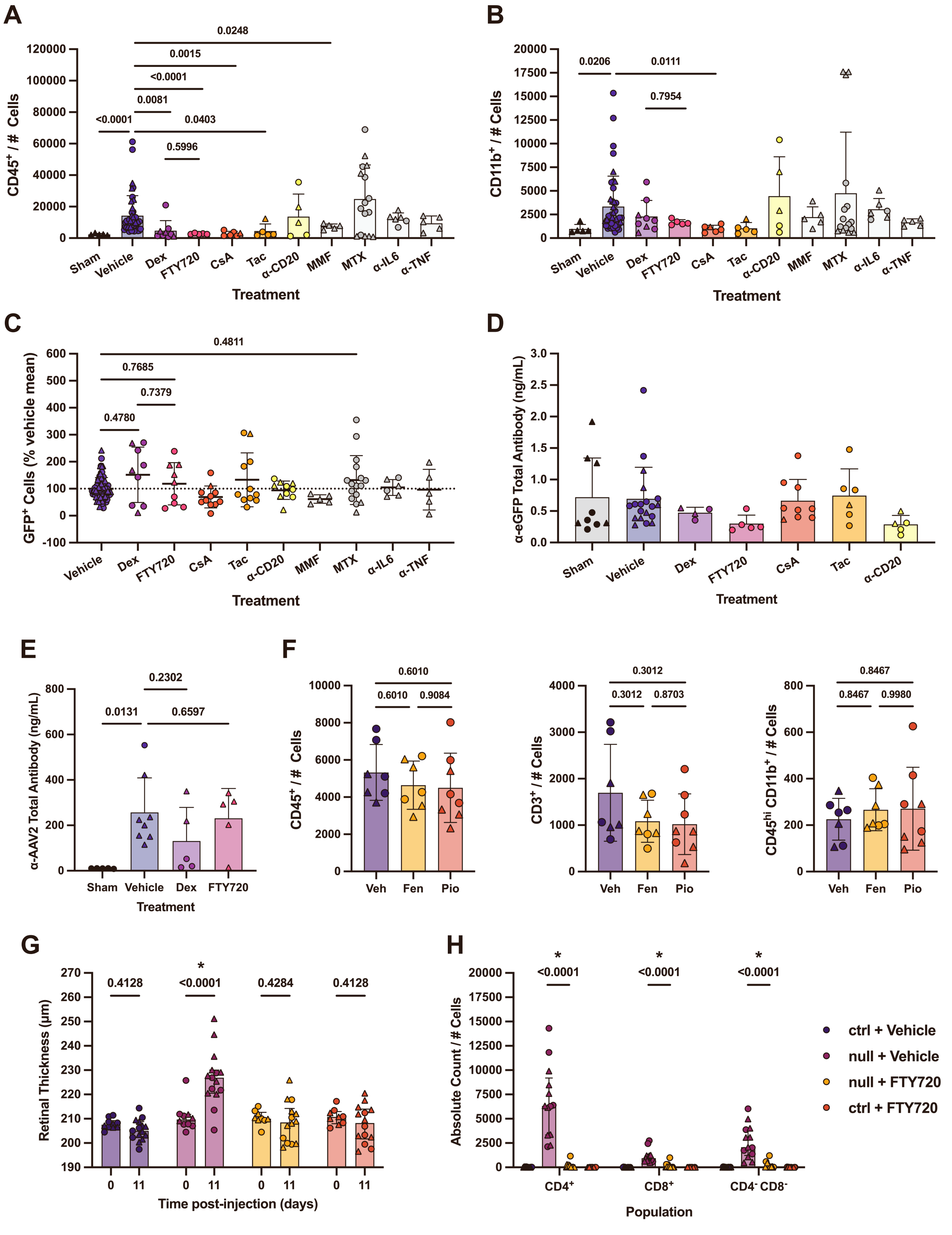

Figure S6. Screening of therapeutic agents to treat rAAV-induced GTAU.

(A-E) Wild-type C57BL/6J mice were treated with fingolimod (FTY720), dexamethasone (Dex), methotrexate (MTX), mycophenolate mofetil (MMF), cyclosporine A (CsA), tacrolimus (Tac), and mouse-specific neutralizing antibodies to tumor necrosis factor (TNF), IL-6, and CD20 or vehicle (PBS) every day from -1 to 28 dpi (For specific dosing regimens and administration routes, see Methods). AAV2.CMV.eGFP:WPRE (5.4E9 gc/eye) or sham (PBS) was intravitreally administered at 0 dpi. Retina, choroid, aqueous, vitreous, and uveal tissue from single eyes were processed into single-cell suspensions and stained with viability dye and fluorescent monoclonal antibodies for flow cytometric analysis at 28 dpi. **(A-C)** Absolute counts of $CD45^+$, $CD11b^+$, and GFP^+ (AAV-transduced) cells. **(D)** Titers of anti-eGFP transgene-binding serum antibodies at 28 dpi. **(E)** Titers of anti-AAV2 capsid-binding serum antibodies at 56 dpi (n = 5-8 per group). **(F)** Absolute counts of $CD45^+$, $CD3^+$, and $CD11b^+$ cells from wild-type C57BL/6J mice receiving AAV2.CMV.null:WPRE.io2 (1E10 gc/eye) and were intraperitoneally treated with fenofibrate (20 mg/kg), pioglitazone (5.3 mg/kg), or vehicle every day from -1 to 28 dpi. Retina and vitreous from single eyes were processed for flow cytometric analysis at 28 dpi. **(G)** Total retinal thickness and **(H)** absolute $CD4^+$, $CD8^+$, and $CD3^+$ $CD4^-$ $CD8^-$ cell counts at 12 dpi, from *Cx3cr1*^{CreER}*:R26-tdTomato*$^{+/-}$ C57BL/6J mice receiving AAV2.CMV.null:WPRE.io2 (1E10 gc/eye) and treated with fingolimod (FTY720; 6mg/kg) or vehicle every other day from -1 to 11 dpi. Statistical analysis was performed by Brown-Forsythe and Welch ANOVA with multiple comparisons testing by False Discovery Rate (FDR) correction using the two-stage step-up method of Benjamini, Krieger, and Yekutieli, with q values reported.

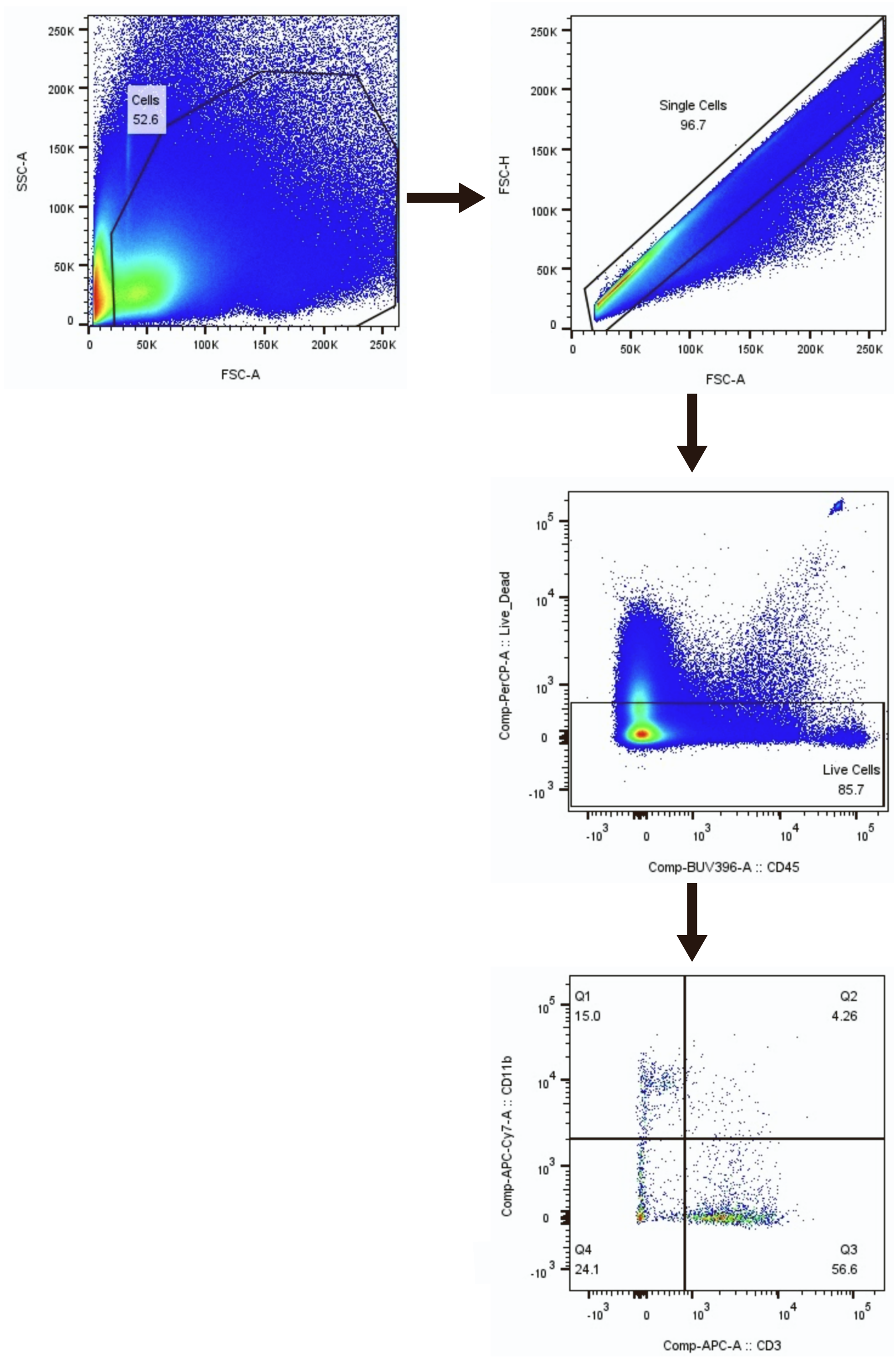

Figure S7. Whole-eye C57BL/6J wild-type mouse flow cytometry gating strategy.

Wild-type C57BL/6J mice were intravitreally injected with AAV2.CMV.eGFP:WPRE (5.4E9 gc/eye) at 0 days post-injection (dpi). Retina, choroid, aqueous, vitreous, and uveal tissue from single eyes were processed into single-cell suspensions and stained with viability dye and fluorescent monoclonal antibodies for flow cytometric analysis at 28 dpi. Samples were gated for cells, singlets, live cells, and immune cell subtypes. Fluorescence channel spillover was corrected in BD FACSDiva by compensation using single-stain controls. Data was acquired on a BD FACSymphony A3 Cell Analyzer and analyzed in FlowJo 10.8.1.

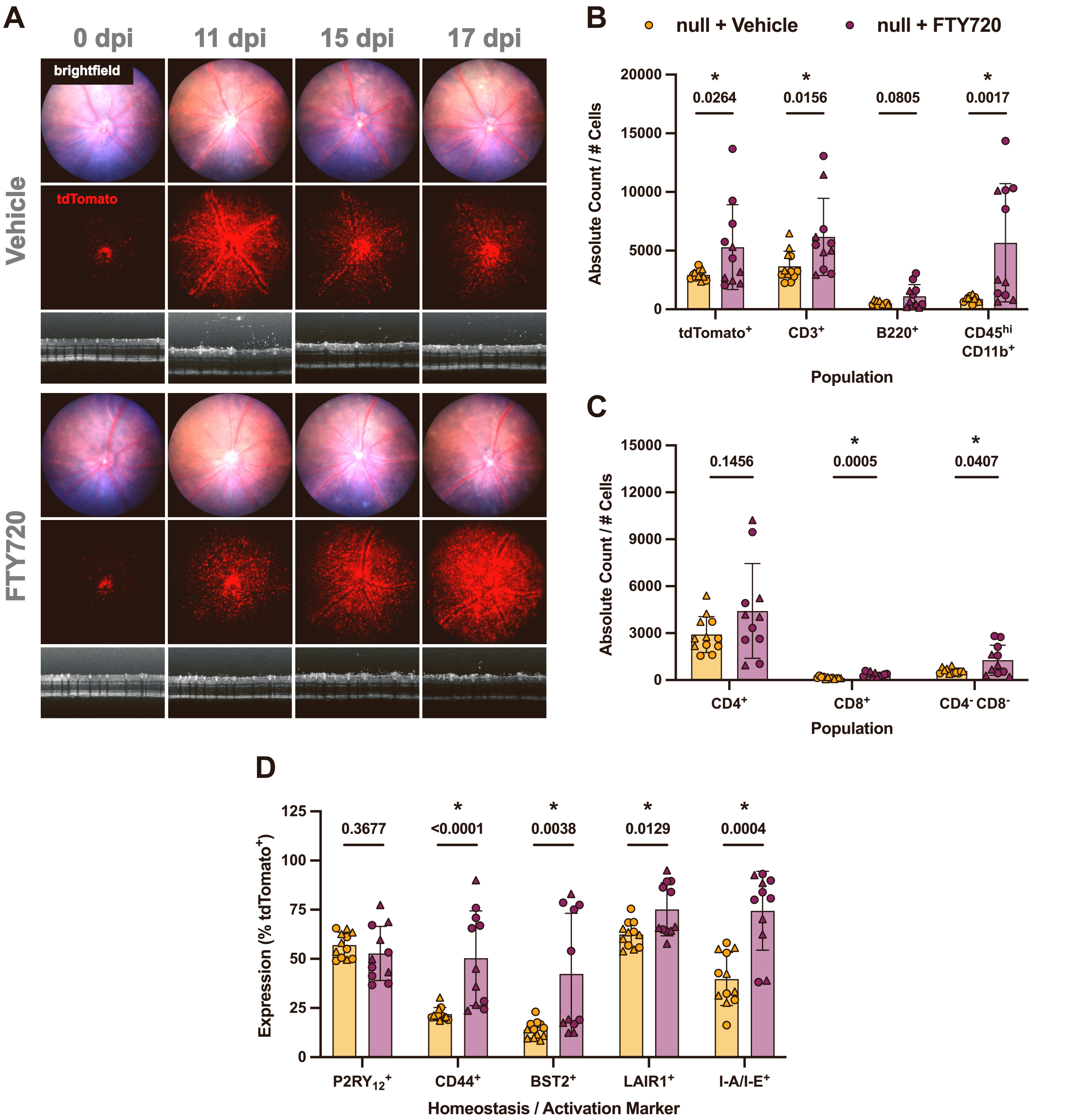

Figure S8. Withdrawal of FTY720 treatment at 11 dpi causes inflammation by 18 dpi.

Cx3cr1^CreER^:R26-tdTomato$^{+/-}$ C57BL/6J mice receiving AAV2.CMV.null:WPRE.io2 (1E10 gc/eye) were orally treated with fingolimod (FTY720; 6mg/kg) or vehicle (ddH_2O with 0.167% v/v DMSO) every other day from -1 to 11 dpi. Retina and vitreous from single eyes were processed into single-cell suspensions, stained, and analyzed by multicolor flow cytometry at 18 dpi. **(A)** Representative clinical and subclinical images (fundus brightfield, tdTomato fluorescence, OCT circle scan) captured at 0, 11, 15, and 17 dpi by in vivo Micron IV imaging. **(B)** Absolute counts of resident and infiltrating CD45$^+$ cells in the posterior compartment at 18 dpi (n = 11-12 per group). **(C)** Absolute counts of CD3$^+$ cell subsets in the posterior compartment at 18 dpi (n = 11-12 per group). **(D)** Protein expression of selected microglial homeostasis and activation markers on tdTomato$^+$ cells at 19 dpi (n = 11-12 per group). Statistical analysis was performed by Welch t-test or Mann-Whitney test, with p values reported. * indicates p ≤ 0.05. Columns and error bars represent mean ± SD, respectively.

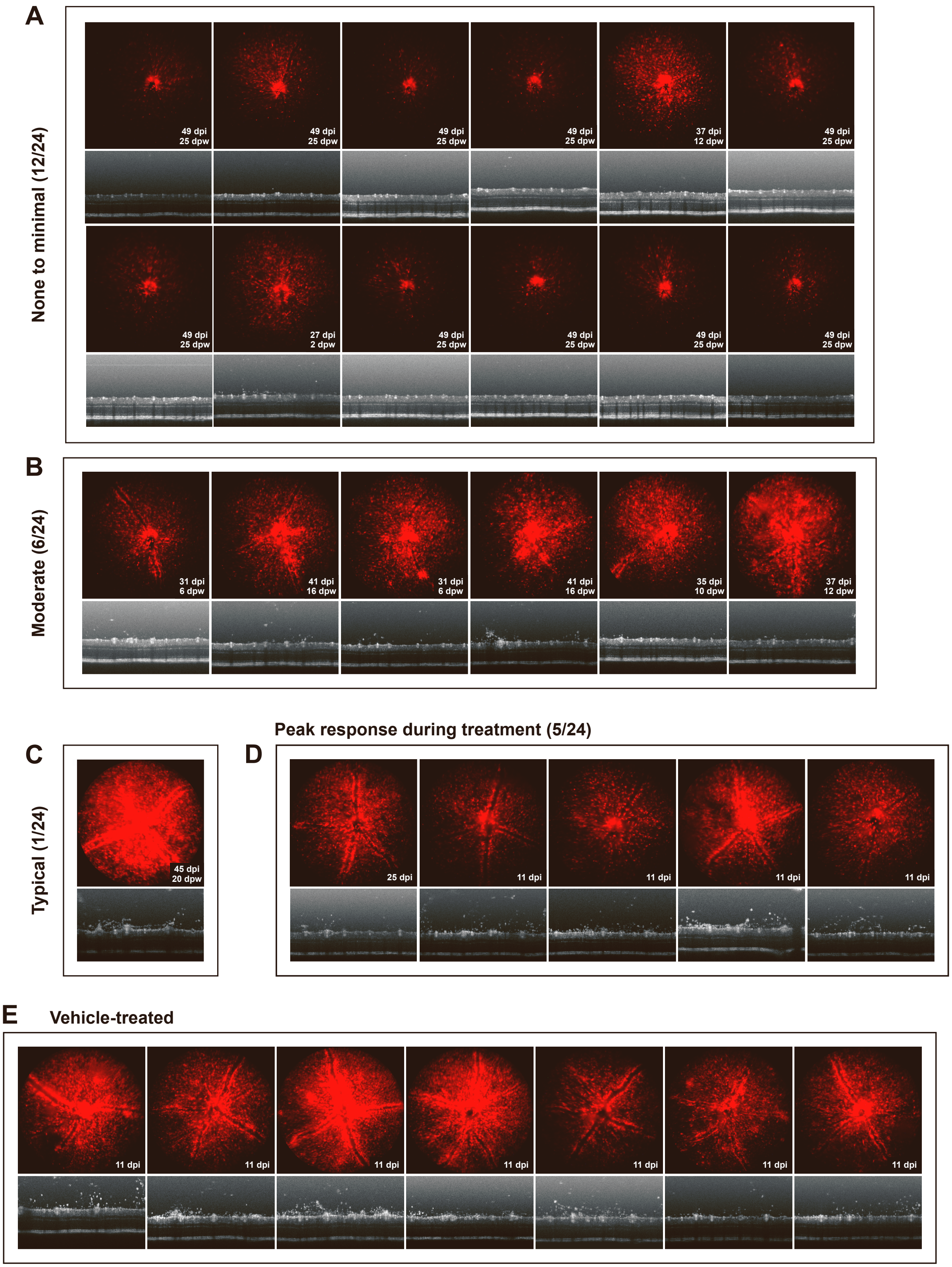

Figure S9. Maximal response phenotypes of vehicle and fingolimod-treated animals during extended treatment withdrawal experiments. *Cx3cr1*^{CreER}*:R26-tdTomato*$^{+/-}$ C57BL/6J mice receiving AAV2.CMV.null:WPRE.io2 (1E10 gc/eye) were orally dosed with fingolimod (FTY720; 6 mg/kg) or vehicle (ddH$_2$O with 0.167% v/v DMSO) every other day from -1 to 25 dpi. Mice were imaged at regular intervals until 49 dpi by *in vivo* Micron IV imaging. To illustrate variation in the presence and severity of inflammation, maximal response phenotypes of tdTomato fluorescence and vitritis throughout the withdrawal period (25 to 49 dpi) from clinical images were qualitatively classified: **(A)** No evidence or minimal extent of clinical inflammation (vitritis) and microglial activation (elevated fluorescence or altered distribution); **(B)** Moderate responses with limited vitritis and focal perivascular accumulation of tdTomato$^+$ cells; **(C)** Typical GTAU with characteristic vitritis and widespread perivascular accumulation of tdTomato$^+$ cells; **(D)** Immune responses of varying intensity developed during the treatment period to 25 dpi despite FTY720 administration. To facilitate comparison, representative maximal responses of vehicle-treated animals are provided in **(E)**. For each eye, day post-injection (dpi) and day post-withdrawal (dpw) are indicated.

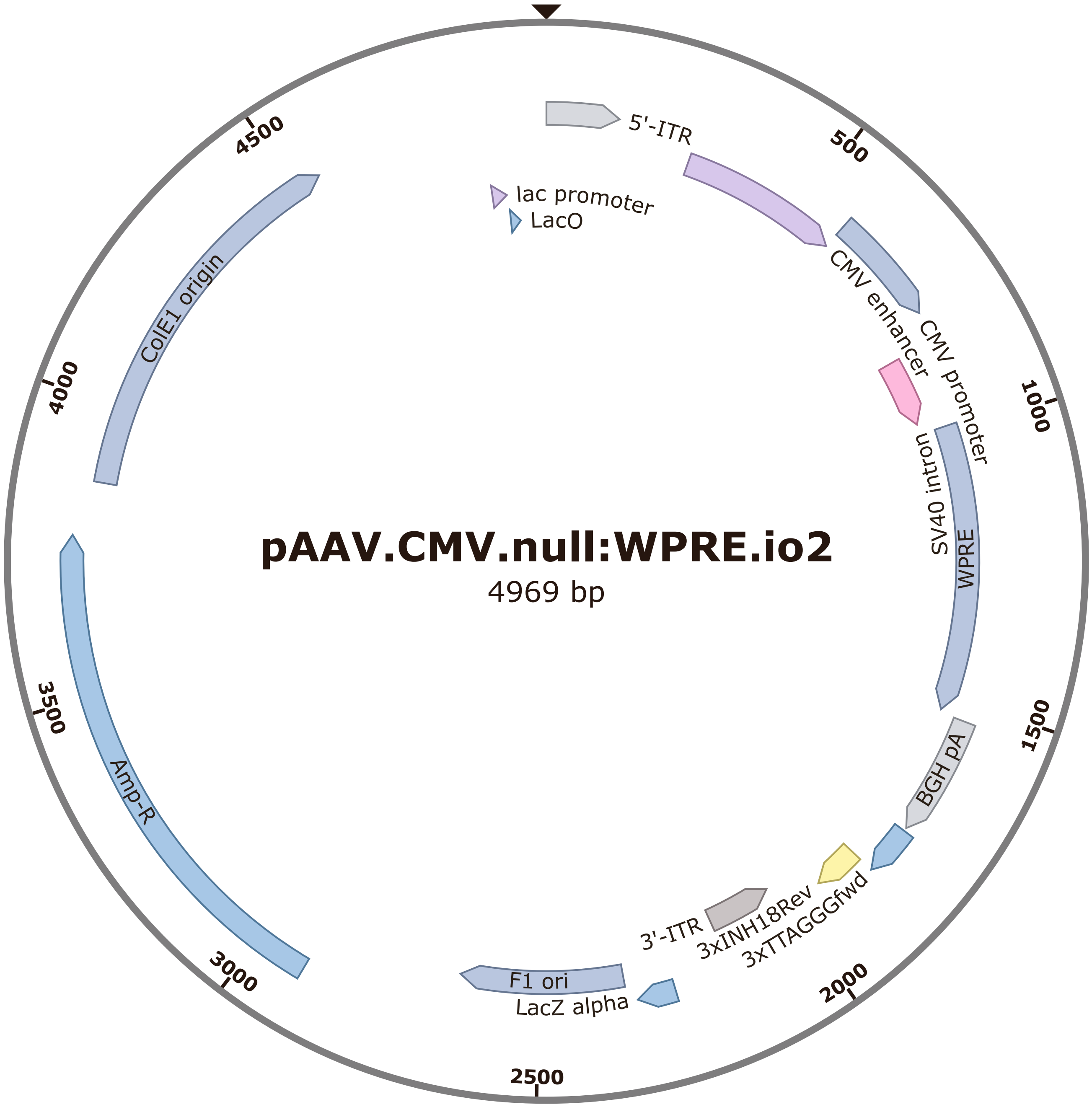

Figure S10. Visual map of pAAV.CMV.null:WPRE.io2.

Vector sequence incorporated between 5' and 3' inverted terminal repeats (ITRs), comprising cytomegalovirus (CMV) enhancer and promoter, simian virus 40 (SV40) intron, Woodchuck Hepatitis Virus post-transcriptional regulatory element (WPRE), and a bovine growth hormone (bGH) polyadenylation (polyA) sequence. TLR9-inhibiting io2 sequence is annotated in two parts, '3xINH18Rev' and '3xTTAGGGfwd'. Numeric annotation along the circumference denotes nucleotide position from the 5' ITR sequence start. Visualisation generated using Benchling (Benchling, San Francisco, CA, USA).

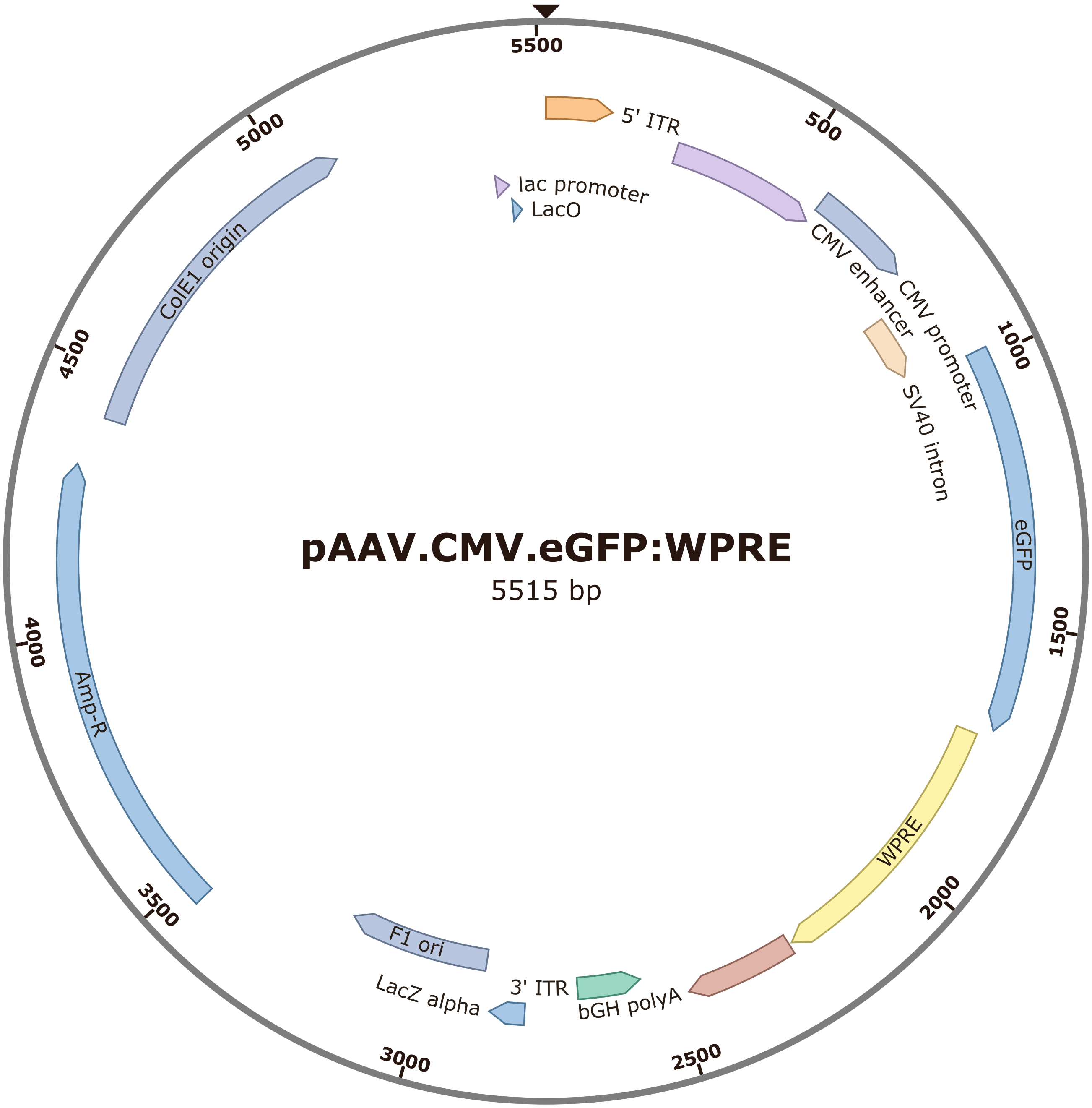

Figure S11. Visual map of pAAV.CMV.eGFP:WPRE.

Vector sequence incorporated between 5' and 3' inverted terminal repeats (ITRs), comprising cytomegalovirus (CMV) enhancer and promoter, simian virus 40 (SV40) intron, enhanced green fluorescent protein (eGFP), Woodchuck Hepatitis Virus post-transcriptional regulatory element (WPRE), and a bovine growth hormone (bGH) polyadenylation (polyA) sequence. Numeric annotation along the circumference denotes nucleotide position from the 5' ITR sequence start. Visualisation generated using Benchling (Benchling, San Francisco, CA, USA).

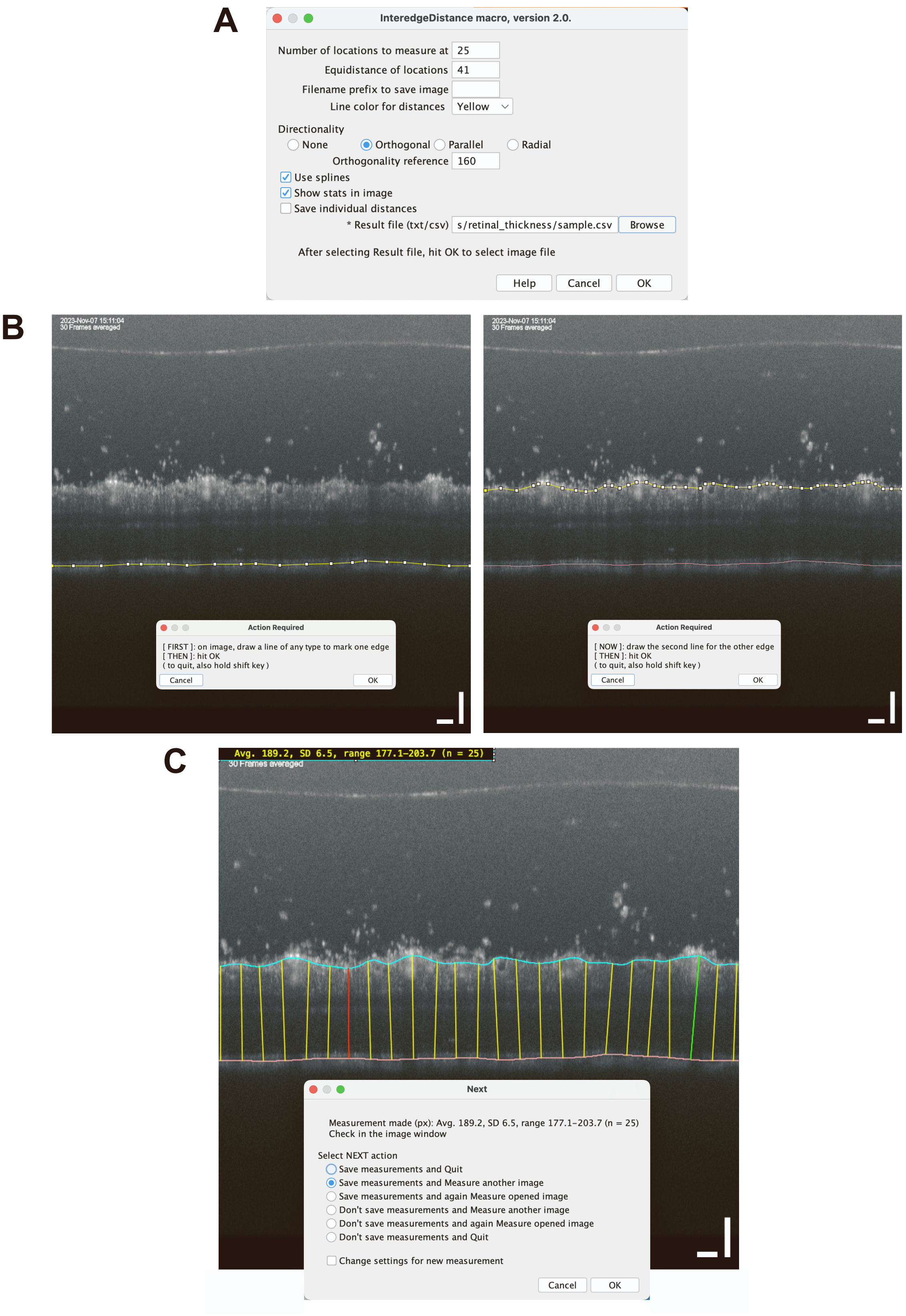

Figure S12. Quantification of inflammation-induced retinal edema by human-guided machine measurement of total retinal thickness. **(A)** Parameters for thickness measurement, including the number of measurements (n = 25), distance between measurements (in pixels; horizontal image size of 1024 px divided by 25 measurements), directionality of measurement as orthogonal (90°), and orthogonality reference as approximate distance between retinal interfaces (in px). **(B)** Human-defined retinal interfaces, specifically the internal limiting membrane (ILM) and Bruch's membrane (BM). **(C)** Quantification of ILM–BM distance (including mean, standard deviation, and range in px), with each of 25 measurements illustrated as a yellow line. Shortest and longest measurements highlighted in red and green, respectively. Measurements in px were subsequently converted via the 100 µm vertical scale bar (78 px) according to the following formula: Retinal thickness (µm) = mean measurement (px) ÷ 78 × 100.

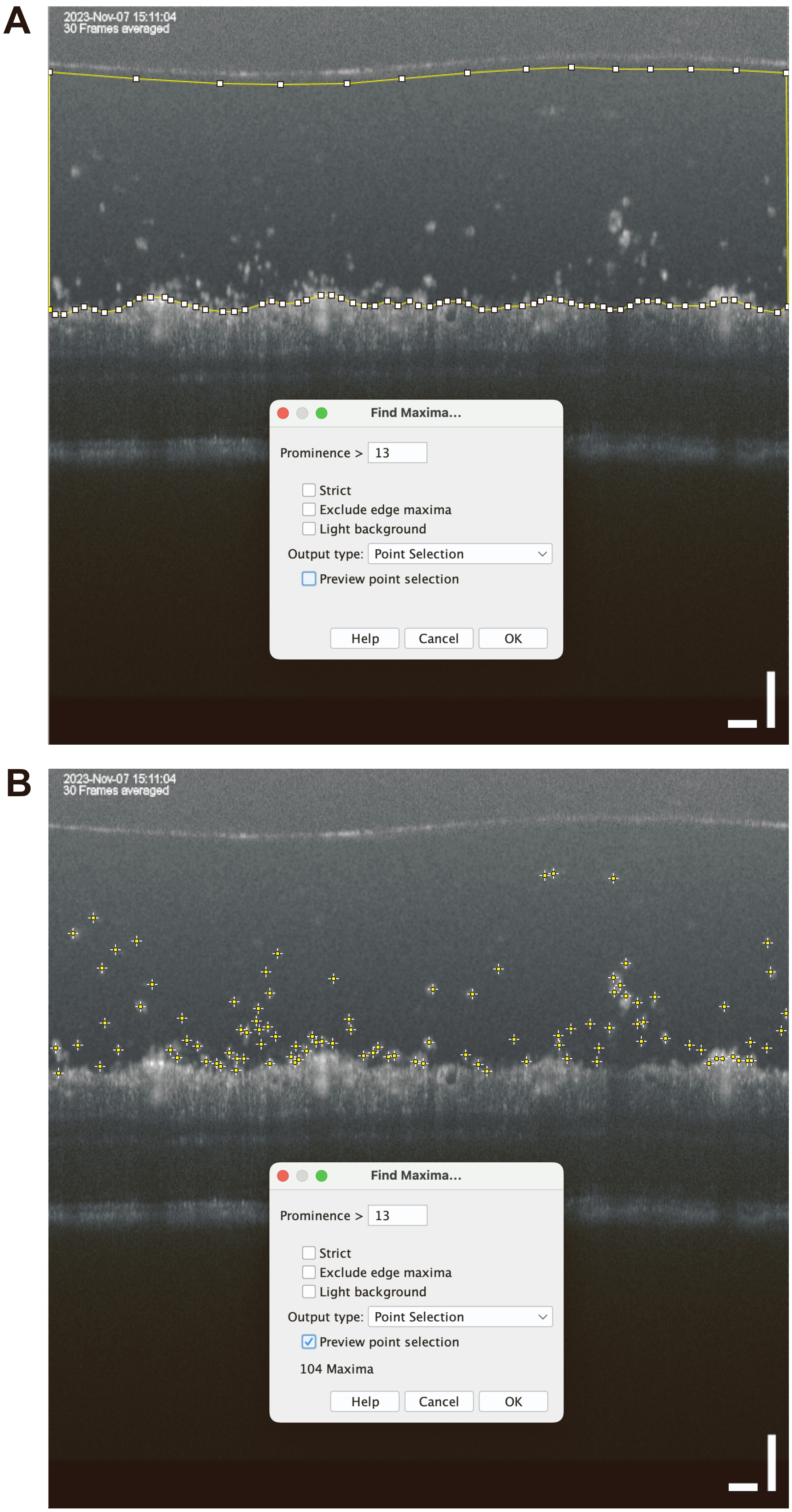

Figure S13. Quantification of vitritis by human-guided machine identification of cellular infiltrate.
(A) The vitreous body is defined by a polygon drawn by a human investigator blinded to treatment conditions from the inner limiting membrane (ILM) to the lens using the 'Polygon Selection' tool in ImageJ. **(B)** Within this polygon, vitritis is machine-quantified by the 'Find Maxima' function in ImageJ, using a prominence value in the range of 13–18. To ensure reliability, machine-based identification was confirmed by a human investigator.

Supplementary Information: Captions for Tables S1-S26

Table S1. Differential gene expression analysis between samples of 3-month-old FACS-isolated retinal microglia at 12 and 0 days post-injection.

Table S2. Differential gene expression analysis between samples of 3-month-old FACS-isolated retinal microglia at 28 and 12 days post-injection.

Table S3. Differential gene expression analysis between samples of 3-month-old FACS-isolated retinal microglia at 28 and 0 days post-injection.

Table S4. Read quantification of genes expressed in samples of 3-month-old FACS-isolated retinal microglia at 0 dpi as transcripts per million (TPM)

Table S5. Read quantification of genes expressed in samples of 3-month-old FACS-isolated retinal microglia at 12 dpi as transcripts per million (TPM).

Table S6. Read quantification of genes expressed in samples of 3-month-old FACS-isolated retinal microglia at 28 dpi as transcripts per million (TPM).

Table S7. Over-representation analysis of 3-month-old FACS-isolated retinal microglia samples at 12 vs. 0 days post-injection via KEGG pathways reveals functional insights.

Table S8. Over-representation analysis of 3-month-old FACS-isolated retinal microglia samples at 12 vs. 0 days post-injection via Gene Ontology Biological Process terms reveals functional insights.

Table S9. Over-representation analysis of 3-month-old FACS-isolated retinal microglia samples at 12 vs. 0 days post-injection via Gene Ontology Cellular Component terms reveals functional insights.

Table S10. Over-representation analysis of 3-month-old FACS-isolated retinal microglia samples at 12 vs. 0 days post-injection via Gene Ontology Molecular Function terms reveals functional insights.

Table S11. Gene Set Enrichment Analysis (GSEA) of 3-month-old FACS-isolated retinal microglia samples at 12 vs. 0 days post-injection via KEGG pathways reveals functional insights.

Table S12. Gene Set Enrichment Analysis (GSEA) of 3-month-old FACS-isolated retinal microglia samples at 12 vs. 0 days post-injection via Gene Ontology Biological Process terms reveals functional insights.

Table S13. Gene Set Enrichment Analysis (GSEA) of 3-month-old FACS-isolated retinal microglia samples at 12 vs. 0 days post-injection via Gene Ontology Cellular Component terms reveals functional insights.

Table S14. Gene Set Enrichment Analysis (GSEA) of 3-month-old FACS-isolated retinal microglia samples at 12 vs. 0 days post-injection via Gene Ontology Molecular Function terms reveals functional insights.

Table S15. Analysis-Ready Molecules for Ingenuity Pathway Analysis from 3-month-old FACS-isolated retinal microglia samples at 12 vs. 0 days post-injection.

Table S16. Ingenuity Pathway Analysis of 3-month-old FACS-isolated microglia samples at 12 vs. 0 days post-injection reveals dysregulated Canonical Pathways.

Table S17. Ingenuity Pathway Analysis of 3-month-old FACS-isolated microglia samples at 12 vs. 0 days post-injection reveals potential Upstream Regulators.

Table S18. Ingenuity Pathway Analysis of 3-month-old FACS-isolated microglia samples at 12 vs. 0 days post-injection reveals potential Causal Networks.

Table S19. Analysis-Ready Molecules for Ingenuity Pathway Analysis from 3-month-old FACS-isolated retinal microglia samples at 28 vs. 12 days post-injection.

Table S20. Ingenuity Pathway Analysis of 3-month-old FACS-isolated microglia samples at 28 vs. 12 days post-injection reveals dysregulated Canonical Pathways.

Table S21. Ingenuity Pathway Analysis of 3-month-old FACS-isolated microglia samples at 28 vs. 12 days post-injection reveals potential Upstream Regulators.

Table S22. Ingenuity Pathway Analysis of 3-month-old FACS-isolated microglia samples at 28 vs. 12 days post-injection reveals potential Causal Networks.

Table S23. Analysis-Ready Molecules for Ingenuity Pathway Analysis from 3-month-old FACS-isolated retinal microglia samples at 28 vs. 0 days post-injection.

Table S24. Ingenuity Pathway Analysis of 3-month-old FACS-isolated microglia samples at 28 vs. 0 days post-injection reveals dysregulated Canonical Pathways.

Table S25. Ingenuity Pathway Analysis of 3-month-old FACS-isolated microglia samples at 28 vs. 0 days post-injection reveals potential Upstream Regulators.

Table S26. Ingenuity Pathway Analysis of 3-month-old FACS-isolated microglia samples at 28 vs. 0 days post-injection reveals potential Causal Networks.

Table S27. Selected gene and protein markers of microglia homeostasis and activation.

Collection of gene and protein marker pairs indicating the microglial state of homeostasis or activation, based on previous research and analysis of transcriptomic data obtained from the Gene Expression Omnibus repository (GSE266332).[20] Genes were grouped into modules based on function.

Module	Protein	Gene	Localization	Function
Homeostasis	P2RY$_{12}$	*P2ry12*	Surface	Surveillance of neuronal health and function by ADP chemoreception[38]
Activation and migration	BST2	*Bst2*	Surface	Inhibition of viral budding, activation of NF-κB[39]
	CD44	*Cd44*	Surface, secreted	Cell adhesion and migration via binding to extracellular matrix[40]
Antigen presentation	H2-Kb	*H2-K1*	Surface	Presentation of intracellular antigens to $CD3^+$ $CD8^+$ cytotoxic T lymphocytes (MHC Class I)[41]
	I-A/I-E	*H2-Aa*	Surface	Presentation of extracellular antigens to $CD3^+$ $CD4^+$ helper and regulatory T lymphocytes (MHC Class II)[41]
Suppression	Qa-2	*H2-Q7*	Surface, secreted	Inhibition of activation and proliferation of NK and T cells (MHC Class I)[42,43]
	LAIR1	*Lair1*	Surface, secreted	Inhibition of myeloid activation and proliferation, C1q and collagen receptor[44,45]

Table S28. Summary of differences in vector production for AAV used in University of Bristol (Figures 1-5, 6C-F, 7A-D) and University of Washington (Figures 6A&B, 7E-G) laboratories.

	University of Bristol	University of Washington
**Manufacturer**	VectorBuilder	UMass Viral Vector Core
**Production system**	Triple transfection HEK293T	Triple transfection HEK293T
**Purification system**	PEG precipitation & CsCl gradient centrifugation	PEG precipitation & CsCl gradient centrifugation
**Titration methodology**	qPCR (ITRs)	ddPCR (CMV)
**Sterility check**	Inoculation into culture medium	Not reported
**Mycoplasma check**	Indicator cell culture	PCR
**Endotoxin check**	LAL testing (< 10 EU/mL)	< 0.5 EU/mL
**Typical full capsid ratio**	Typically > 70%	Not reported
**Storage buffer**	PBS + 0.001% F-68	PBS + 5% D-sorbitol + 0.001% F-68

Table S29. Summary of differences in AAV administration technique used in University of Bristol (Figures 1-5, 6C-F, 7A-D) and University of Washington (Figures 6A&B, 7E-G) laboratories.

	University of Bristol	University of Washington
**Vector name**	AAV2.CMV.null:WPRE.io2	AAV2.CMV.eGFP:WPRE
**Vector dose**	1.00E+10	5.40E+09
**Injected volume (μL)**	2	1.2
**Injection technique**	Direct	Guide hole (30 G)
**Needle gauge**	33	34
**Injection speed**	Slow, manual (Hamilton syringe)	Slow, automated (microinjector; 40nL/sec)
**Monitoring**	Direct under cover glass	Indirect via fluorescein
**Anaesthesia**	Nebulised isoflurane	Injected ketamine/xylazine
**Dilation induction**	Tropicamide + Phenylephrine	Phenylephrine
**Hydration**	Viscotears	Genteal
**Perioperative antimicrobials**	Chloramphenicol	Povidone iodine
**Analgesic**	None	Tetracaine (topical, pre-operative)<br>Paracetamol (30 days)

Table S30. Monoclonal Antibodies, Viability Dyes, and Fluorescent Proteins used for Flow Cytometry in Figures 1-7; S1-2,5-6 & 8.

Target	Clone	Species/Isotype	Conjugate	Supplier	Cat. No.
CD16/32	2.4G2	Rat IgG2b, κ	-	BD Biosciences	553142
B220	RA3-6B2	Rat IgG2a, κ	BV510	BioLegend	103248
BST2	eBio927	Rat IgG2b, κ	PE-eFluor610	eBioscience	61-3172-82
CD11b	M1/70	Rat IgG2b, κ	APC/Cyanine7	BioLegend	101226
CD3ε	145-2C11	Armenian Hamster IgG	BV785	BioLegend	100355
CD3	17A2	Rat IgG2b, κ	APC	BioLegend	100236
CD4	RM4-5	Rat IgG2a, κ	BV650	BioLegend	100555
CD44	IM7	Rat IgG2b, κ	SuperBright 600	Invitrogen	63-0441-82
CD45	30-F11	Rat IgG2b, κ	PE/Cyanine7	BioLegend	103114
CD45	30-F11	Rat IgG2b, κ	BUV395	BD Biosciences	564279
CD8β	H35-17.2	Rat IgG2b, κ	BUV395	BD Biosciences	740278
DAPI	-	-	-	BD Biosciences	564907
Gr-1	RB6-8C5	Rat IgG2b, κ	Alexa Fluor 700	BioLegend	108422
H2-Kb	AF6-88.5	Mouse IgG2a, κ	PerCP/Cyanine5.5	BioLegend	116516
I-A/I-E	M5/114.15.2	Rat IgG2b, κ	BV711	BioLegend	107643
LAIR1	113	Armenian Hamster	PE/Cy5.5	Novus	NBP1-43324PECY55
P2RY12	S16007D	Rat IgG2b, κ	APC	BioLegend	848006
Qa-2	695H1-9-9	Mouse IgG2a, κ	FITC	BioLegend	121710
7-AAD	-	-	-	BioLegend	420404

Table S31. Antibodies and Fluorescent Proteins used for Retinal Flatmount Staining in Figures 4&5.

Target	Clone	Species/Isotype	Conjugate	Supplier	Cat. No.
Biotin	-	Streptavidin	eFluor450	eBioscience	48-4317-82
CD3ε	17A2	Rat IgG2b, κ	Alexa Fluor 647	BioLegend	100209
IBA1	Polyclonal	-	Purified	Fujifilm Wako	019-19741
IB4	-	Rabbit	Biotin	Sigma-Aldrich	L2140
Rabbit	Polyclonal	Goat	Alexa Fluor 555	Invitrogen	A27039
Rabbit	Polyclonal	Goat	Alexa Fluor 647	Invitrogen	A21244
RFP	Polyclonal	Rabbit IgG	Purified	Rockland	600-401-379
